# High Melt Strength Polypropylene via Reactive Extrusion for Injection-Molded Foams: Performance of Virgin and Recycled Blends

**DOI:** 10.1021/acsomega.5c13372

**Published:** 2026-05-25

**Authors:** Mert Emre Öztoksoy, Ramazan Bedirhan Yıldız, Rumeysa Yıldırım, Mehmet Ali Oral, Güralp Özkoç, Mehmet Kodal

**Affiliations:** † Polymer Science and Technology Graduate Programme, 52980 Kocaeli University, 41001 Kocaeli, Türkiye; ‡ 693007 Ravago Petrochemical Company, 41420 Kocaeli, Türkiye; § Chemical Engineering Department, 52971 Istanbul Technical University, 34467 Istanbul, Türkiye; ∥ Chemical Engineering Department, Kocaeli University, 41001 Kocaeli, Türkiye; ⊥ Department of Chemistry and Chemical Processing Technologies, Hereke Asım Kocabıyık Vocational School, Kocaeli University, 41800 Kocaeli, Türkiye; # Chemistry Department, 469683 Istinye University, 34010 Istanbul, Türkiye; ∇ Xplore Instruments B.V., Sittard 6135 KT, The Netherlands

## Abstract

This research demonstrates an eco-friendly and reproducible method to synthesize high melt strength polypropylene (HMSPP) by reactive extrusion of virgin polypropylene (PP) and recycled polypropylene (rPP) blends with low-density polyethylene (LDPE). In contrast to many previous reactive extrusion studies that primarily focused on low-MFR polypropylene grades for extrusion or thermoforming-based processes, the present study targets high-MFR-HMSPP materials suitable for injection molding applications, particularly injection-molded foams. A peroxide initiator (DHBP) and a polyfunctional coagent, namely trimethylolpropane triacrylate (TMPTA), were added to the blends to induce long-chain branching (LCB) while maintaining adequate processability. Detailed analyses were conducted to evaluate the effects of LDPE (20–40 wt %) and TMPTA (2–5 wt %) on morphology, thermal behavior, rheological response, mechanical performance, and foaming characteristics. Rheological investigations revealed pronounced increases in complex viscosity, storage modulus, and melt elasticity, accompanied by strain-hardening behavior in TMPTA-modified blends, which are widely accepted rheological signatures of long-chain branching rather than extensive gel formation. SEM analyses demonstrated improved interfacial compatibility among reactively modified blends and refined LDPE domain structures following reactive modification. The recycled blend (rPP5) displayed similar foamability, indicating the potential capability of the procedure to upcycle waste PP.

## Graphical abstract

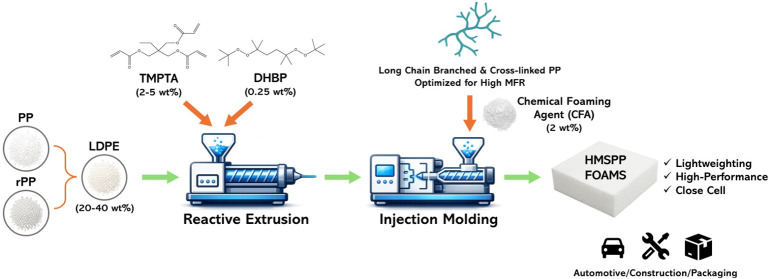

## Introduction

Plastics have spread enormously in usage and applications over the past 40 years in industry and daily life. Because of their large range of applications and processing ease, extensive usage is observed in building, automotive, white goods, packaging, medical devices, and aircraft. A new aspect regarding polymer blend science and technology has also emerged in recent years, as specific applications demand polymeric compositions that can withstand challenging mechanical, chemical, thermal, and electrical conditions.^1^ Polymer-based foamed materials are particularly attractive because of their excellent energy absorption, lightweight, relatively low production cost, and thermal insulation. Among several production techniques, injection molding has become the dominant method for manufacturing designed foam products due to its short molding cycle, high automation, and simplicity of use. In fact, injection molding accounts for almost one-third of global polymer consumption and produces nearly 80% of plastic components.^2–6^

Polypropylene (PP) is one of the semicrystalline thermoplastic polymers and is one of the four major commodity plastics and is found to have wide applications in packaging, textiles, automotive parts, and household items due to its affordability, desirable chemical resistance, and beneficial mechanical properties.^7–10^ However, large-scale manufacturing and usage of PP have also raised sustainability issues. Conventional recycling techniques such as landfilling, energy recovery, and mechanical recycling are widely reported in the literature.^11,12^ Mechanical recycling, in particular, is cost-effective and environmentally friendly, with a theoretical retained value of 20–50% of virgin material market value. Diversified techniques, including the addition of virgin polymers, fillers, fibers, or compatibilizers, have been applied as a means of improving recycled plastic quality. Alternatively, conventional recycling is generally insufficient to fully restore the original performance of PP. For that matter, much attention is focused on the upcycling process, whereby molecular structure is transformed in an attempt to create higher value-added materials with superior performance.^13–22^ Despite all these attempts, traditional PP still suffers from inherent disadvantages. Due to its linear molecular arrangement, it experiences poor melt strength and strain hardening, and thus it sags and is unstable in shaping operations involving extensional flow, including foaming, thermoforming, and blowing.^22–25^

Traditional PP grades, particularly isotactic PP, exhibit low melt elasticity. As a result, they tend to sag and demonstrate instability during shaping operations that demand dimensional integrity under elevated temperature and mechanical stress. Moreover, it is difficult to recycle polypropylene since it possesses relatively low melt strength, and thus used materials are difficult to reprocess without serious degradation. In spite of all these challenges, research is still carried out with the intention of enhancing the characteristics of PP for sustainable production techniques and enhancing recyclability utilizing chemical recycle methods. Furthermore, the relatively low melt strength of PP is still responsible for suppressing reprocessability, and as such, there is potential for recycled PP. The research concerning the brittle-ductile transition of polymers at low temperature also highlights the need for increasing toughness and attaining reproducible service performance since temperature significantly imparts long-term stability.^26–28^ To compensate for these deficiencies, high melt strength polypropylene (HMSPP) was developed. A key characteristic of HMSPP is the presence of long-chain branches (LCBs) that increase zero-shear viscosity, raise melt elasticity, and enable strain-hardening behavior.^29–31^

Several approaches have been developed to introduce branching into polypropylene (PP). Scheve et al.^32^ achieved this by irradiating dense, high-molecular-weight linear PP with high-energy radiation in a nitrogen atmosphere. The resulting irradiated PP exhibited pronounced melt strain hardening. However, irradiation at high energy gives rise to branching of confinement largely in the amorphous region of the structural, semicrystalline PP, since segmental motion and free volume in this region enable macroradicals generated upon irradiation to come near each other and attach and thus give rise to the formation of the branching points. In principle, branching points can even be introduced on the polypropylene backbone through postreactor grafting reactions. Such modifications at the molecular level substantially increase bubble stability in foaming operations and guarantee an added wall thickness in thermoformed parts. HMSPP may be prepared using differing avenues such as in-reactor techniques using the metallocene type of catalysts, postreactor methods involving irradiation, or reactive extrusion (RE). Among these techniques, RE is specifically desirable given that it is a solventless technique, scalable, and one that is amenable and integrated with continuous volumetric manufacturing processes in the plants.^33–38^ In addition to chemical modification, several studies have explored the blending of PP with low-density polyethylene (LDPE) to enhance its melt processability. The incorporation of LDPE contributes to increased melt stretchability and a higher degree of branching. Moreover, LDPE can participate in radical-mediated grafting reactions during reactive extrusion, potentially leading to the formation of cocrosslinked networks that impart improved melt elasticity.^13,39–41^

However, PP/LDPE blends generally exhibit phase separation due to intrinsic incompatibilities arising from differences in their crystalline structures and molecular architectures.^42,43^ Reactive extrusion offers an effective strategy for achieving compatibilization in thermodynamically immiscible polymer blends. In this process, the use of organic peroxides as radical initiators promotes the formation of macroradicals, which may subsequently participate in coupling, disproportionation, or grafting reactions with multifunctional monomers.^44,45^ Lagendijk et al.^46^ prepared long-chain branched polypropylene (LCB-PP) via reactive extrusion employing peroxydicarbonates with differing structural configurations. Nevertheless, PP exhibits a strong tendency toward β-scission, which impedes grafting and cross-linking reactions, particularly when only peroxide initiators are present in the system. The addition of polyfunctional monomers has been shown to suppress degradation and promote a higher degree of long-chain branching.^47–50^ Trimethylolpropane triacrylate (TMPTA), a representative trifunctional acrylate monomer, can be employed as a coagent to enhance the efficiency of branching and network formation control. Kim and Kim^51^ utilized TMPTA to mitigate chain scission and disproportionation side reactions associated with peroxide initiation, thereby diminishing excessive cross-linking. Conversely, Su and Huang^52^ observed that the inclusion of TMPTA in PP systems facilitated the formation of increased long-chain branching. Therefore, in the presence of coagents, reactive extrusion not only facilitates the formation of long-chain branching and minimizes gel formation but also improves the miscibility between PP and LDPE, leading to the production of high-melt-strength PP (HMSPP) blends with superior structural integrity and mechanical performance.^46,53–56^

Building upon these findings, subsequent studies have investigated the combined effects of peroxide type, coagent structure, and blending methodology on phase morphology, strain-hardening behavior, and foamability. Although these investigations have yielded promising results, most lack comprehensive comparisons between foam performance and morphological evolution that explicitly correlate foamability with structural transformations. Notably, there remains a scarcity of research addressing industrial applications such as injection molding, which typically employ materials with high melt flow rate values, as well as studies comparing neat and recycled polymers. This gap underscores the need for systematic investigations that elucidate the relationship between foamability and molecular architecture in practical PP/LDPE and recycled polypropylene rPP/LDPE blend systems.

In this study, a systematic approach was presented for the production of high melt strength polypropylene via reactive extrusion of PP/LDPE and rPP/LDPE blends in the presence of TMPTA and peroxide. The effects of LDPE and TMPTA content on morphological features, crystallinity, rheological behavior, and foaming performance are systematically investigated to elucidate the mechanisms underlying structure development and melt strength enhancement. Rather than introducing a fundamentally new chemical modification route, the contribution of this work lies in the systematic evaluation of high MFR HMSPP grades suitable for injection molding applications and the direct comparison between virgin and recycled PP systems under identical reactive extrusion conditions. Unlike many previous studies primarily focused on virgin PP, this work emphasizes the utilization of rPP and LDPE, addressing sustainability challenges while achieving enhanced foaming performance. Furthermore, the correlating morphological and rheological evolution with quantitative foaming metrics revealed cell densities up to 1.1 × 10^8^ cells/cm^3^ and reduced average cell sizes of 7.48 μm. This study demonstrates structure–property-processing correlations within the investigated formulation range. These findings provide a foundation for optimizing scalable production of high-performance HMSPP materials, with potential applications in lightweight automotive components, energy-efficient packaging, and durable building materials.

## Experimental Section

### Materials

Homo polypropylene (PPH, melt flow rate, MFR = 23 g/10 min) was supplied by Rom Petrochemical Company, while low-density polyethylene (LDPE, MFR = 22 g/10 min) was obtained from Petkim Petrochemical Company. Recycled homo polypropylene (rPP, MFR = 25 g/10 min) in granulate form was sourced from postindustrial scrap provided by Ravago Petrochemical Company. Trimethylolpropane triacrylate (TMPTA, Arkema) and 2,5-dimethyl-2,5-(*tert*-butylperoxy)­hexane (DHBP, Nouryon) were used as received without further purification. DHBP concentration was kept constant as 0.25 wt %. TMPTA was incorporated as a coagent at concentrations of 2 and 5 wt % to enhance the mechanical performance of the blends, whereas LDPE was added at 20 and 40 wt % to promote branching through radical formation induced by peroxide decomposition. The chemical blowing agent (CFA) employed in this study was Hydrocerol ITP 825 (Avient). Upon endothermic activation at 200 °C, the sodium bicarbonate (NaHCO_3_) component decomposes, releasing carbon dioxide (CO_2_) into the injection molding barrel. All materials and reagents used were of high purity and conformed to reagent-grade specifications.

### Preparation of Samples

All samples were processed using a Cooperation Brand 26 twin-screw extruder, equipped with K-Tron feeders for material input. The extruder features a 26 mm screw diameter, a production capacity of 60 kg/h, and a maximum barrel temperature of 450 °C. The screw configuration was specifically designed to provide both effective dispersive and distributive mixing while ensuring adequate residence time for the reactions. Barrel temperatures from the feed zone to the die zone were set sequentially as follows: 190, 215, 230, 230, 225, 220, 210, 215, 200, 200, and 220 °C. Table 1 shows the compositions of PP/LDPE and rPP/LDPE blends prepared in this study.

**Table 1:** Compositions of PP/LDPE and rPP/LDPE Blends

sample codes	PP (%)	rPP (%)	DHBP (%)	TMPTA (%)	LDPE (%)
**PP0**	100		0	0	0
**PP1**	99.75		0.25	0	0
**PP2**	80		0	0	20
**PP3**	60		0	0	40
**PP4**	77.75		0.25	2	20
**PP5**	57.75		0.25	2	40
**PP6**	74.75		0.25	5	20
**PP7**	54.75		0.25	5	40
**rPP0**		100	0	0	0
**rPP3**		60	0	0	40
**rPP5**		57.75	0.25	2	40
**rPP7**		54.75	0.25	5	40

Following compounding, the resulting products were fabricated into test specimens using an Engel injection molding machine in accordance with ISO 527–2/1A and ISO 180 standards for mechanical testing. Foamed samples were also prepared using the same injection molding equipment. In this case, dry blends containing 2 wt % chemical foaming agent (CFA) were injected into the ISO 180 mold and foamed in situ under the following processing conditions: a melt temperature profile of 190–210–205–190 °C, a mold temperature of 40 °C, an injection speed of 138.9 mm/s, an injection time of 1.15 s, a cooling time of 12 s, a plasticizing time of 1.15 s, and a maximum injection pressure of 200 bar (actual pressure at filling: 21.7 bar). No holding or packing pressure was applied.^57^

### Morphological and Structural Characterization of Samples

Fourier transform infrared spectroscopy with attenuated total reflection (FTIR-ATR; PerkinElmer Spectrum Two) was performed over the wavenumber range of 4000–650 cm^–1^ to characterize the chemical structures and potential intermolecular interactions.

Scanning electron microscopy (SEM) was employed to analyze the morphology and foaming behavior of PP5, PP7, rPP5, and rPP7, as well as their foamed counterparts. SEM images were obtained from cryogenically fractured surfaces of the impact specimens. Prior to imaging, the samples were coated with a thin layer of gold to prevent charging.

The diameters of microcells observed in SEM images were measured using ImageJ software. The average cell diameter (*D*_0_) was calculated from three distinct regions of each image according to eq 1.

1 $$D_{0}=\frac{\Sigma d_{i}n_{i}}{n_{i}}$$

Where *n*_*i*_ represents the number of cells with a perimeter-equivalent diameter *d*_*i*_ for *i* > 100, eq 2 is used to calculate the cell density (*N*_0_), defined as the number of cells per unit volume of the foamed material.

2 $$N_{0}={\left(\frac{n}{A}\right)}^{3/2}\Phi$$

where *n* is the number of cells counted in the SEM image and *A* is the area of the micrograph (cm^2^). The exponent 3/2 accounts for the conversion from two-dimensional micrograph data to three-dimensional cell density, assuming uniform spherical cell distribution. eq 3 was used to determine the volume expansion ratio (Φ).

3 $$\Phi =\frac{p_{s}}{p_{f}}$$

The density of the foamed samples (*p*_f_) and the density of the unfoamed samples (*p*_s_) were measured according to the ISO1183 1-A standard.^58^

Rheological measurements were conducted using a stress-controlled MCR 102 Anton Paar rotational rheometer equipped with parallel plates on samples approximately 2 mm in thickness. During testing, nitrogen gas was employed to purge the heating chamber. Angular frequency sweep tests were performed at a constant temperature of 190 °C. The measurements included an angular frequency range of 0.1–600 rad/s at a shear strain of 1%.

Tensile properties of the samples were evaluated at room temperature following a minimum conditioning period of 24 h to ensure complete postcrystallization, in accordance with ISO 527, using a ZWICK universal testing machine at a crosshead speed of 50 mm/min. Izod impact tests, without notches, were carried out using the same ZWICK testing apparatus in accordance with ISO 180/1A. For each sample group, a minimum of six specimens were tested, and the results were reported as mean values with corresponding standard errors.

Differential scanning calorimeter (DSC, Mettler Toledo DSC3 Star model) was used to examine the samples thermal characteristics. In a nitrogen atmosphere, DSC analyses were performed between −80 and 300 °C at a heating rate of 10 °C/min. Equations 4 and ([Disp-formula eq5]) are used to determine the percentage crystallinity (%χ) of LDPE and PP and in the blends, respectively.

4 $$\%\chi_{\mathrm{LDPE}}=\frac{\Delta H_{m}^{i}}{\varphi_{\mathrm{LDPE}}\Delta H_{m}^{0}\left(\mathrm{LDPE}\right)}\times 100$$

5 $$\%\chi_{\mathrm{PP}}=\frac{\Delta H_{m}^{f}}{\varphi_{\mathrm{PP}}\Delta H_{m}^{0}\left(\mathrm{PP}\right)}\times 100$$

where Δ*H*_m_^i^ is the melting enthalpy of LDPE, Δ*H*_m_^f^ is the melting enthalpy of PP, %χ_PP_ is the percentage crystallinity of PP, %χ_LDPE_ is the percentage crystallinity of LDPE, Δ*H*_m_^0^ indicates the equilibrium melting enthalpy of LDPE = 288 J/g,^59^ Δ*H*_m_^0^ (PP) indicates the equilibrium melting enthalpy of PP = 207 J/g,^60^ φ_PP_ is the weight fraction of PP, and φ_LDPE_ is the weight fraction of LDPE.

### Gel Content Determination

Gel content measurements were performed to evaluate the extent of cross-linking in the reactively modified samples. Approximately 0.7–0.8 g of the extruded granules were processed into 2 mm thick films via compression molding to obtain specimens for subsequent gel content analysis. The films were then cut into small pieces, weighed (*W*_0_), and placed in a thimble. The samples were extracted in boiling xylene using a Soxhlet apparatus for 24 h to dissolve the soluble fraction.^61^ Xylene was selected as a solvent due to its ability to dissolve un-cross-linked polypropylene while preserving the three-dimensional network structure formed through intermolecular cross-linking reactions.

Following extraction, the samples were removed and dried in a vacuum oven at 80 °C for 12 h to eliminate residual solvent. The dried insoluble fraction was weighed (*W*_1_). The gel content (%) was calculated according to eq 6

6 $$\mathrm{gel}\;\mathrm{content}\;\left(\%\right)=\frac{W_{1}}{W_{0}}\times 100$$

where *W*_0_ is the initial weight of the sample and *W*_1_ is the weight of the insoluble fraction after extraction and drying.

All measurements were performed in triplicate, and average values were reported.

## Results and Discussion

### FTIR Results

FTIR-ATR was employed to investigate the chemical characteristics of pure PP, LDPE, and their blends. The spectral data, collected over the wavenumber range of 4000–650 cm^–1^ (Figure 1), highlight the characteristic peaks, their assignments, and the chemical structures of the analyzed components. FTIR spectroscopy effectively revealed the distinct vibrational modes of both polymers. Literature reports indicate that PP exhibits C–H stretching vibrations between 2810–2985 cm^–1^, with bending vibrations of −CH_2_ and −CH_3_ groups appearing at 1440–1475 and 1370–1380 cm^–1^, respectively.^61^

**Figure 1:**
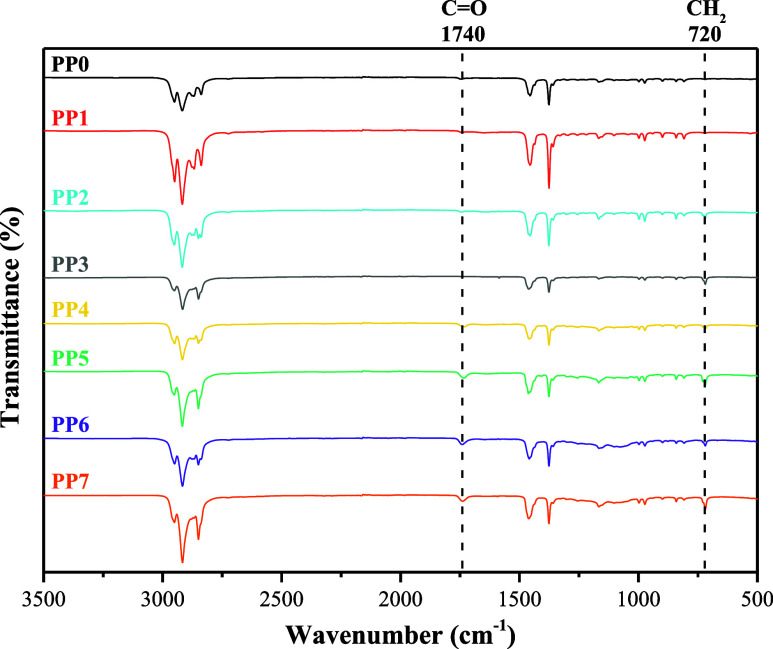
FTIR spectra of pure PP and PP/LDPE blends.

The spectra of PP/LDPE blends appear as a simple superposition of the individual polymer spectra, indicating that the blending process is primarily physical, with no new absorption bands indicative of chemical interactions or covalent bond formation between the PP and LDPE phases (Figure 2). The absorption band located at 720 cm^–1^ is due to the rocking vibration of LDPE in the blends.^62^ A notable finding concerns the role of the trimethylolpropane triacrylate (TMPTA) additive. The appearance of a low-intensity peak around 1740 cm^–1^, corresponding to the carbonyl (C=O) stretching vibration, suggests the possible incorporation of TMPTA-derived carbonyl-containing moieties into the polymer structure. This phenomenon can be attributed to a radical mechanism in which PP chains initially undergo hydrogen abstraction mediated by peroxide free radicals, forming polypropylene macroradicals. These macroradicals subsequently react with TMPTA monomers, potentially leading to the formation of branched structures containing carbonyl fragments within the polymer matrix.^63^

**Figure 2:**
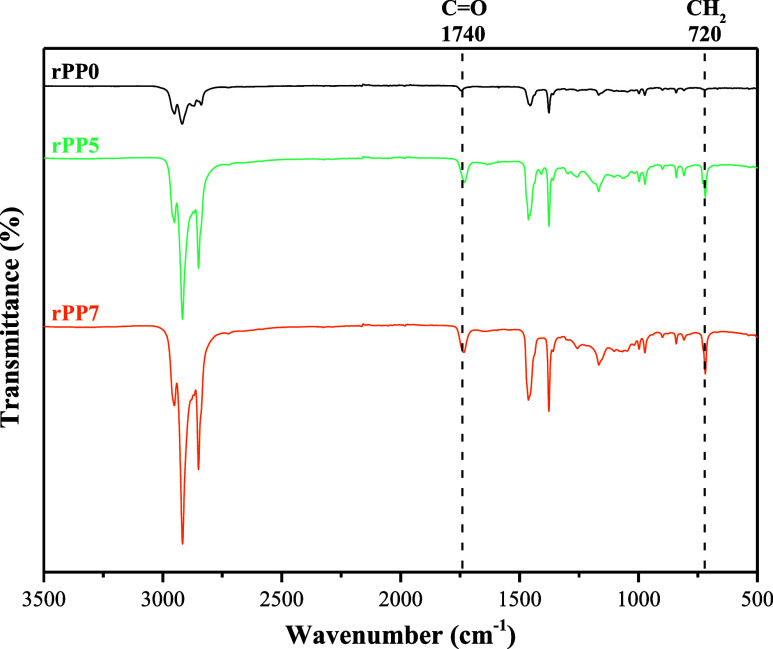
FTIR spectra of rPP and rPP/LDPE blends.

The FTIR analysis results are particularly significant, as they provide critical insights into the chemical integrity of the material during recycling. Comparison of the spectra obtained before and after the recycling process revealed no shifts in the positions of the characteristic peaks, nor the appearance of new bands indicative of oxidative degradation, such as broad hydroxyl or carbonyl absorptions. These observations suggest that, under the applied recycling conditions, no major chemical alterations detectable by FTIR were observed. However, it should be noted that FTIR analysis primarily provides qualitative information, and subtle molecular weight changes or limited oxidative degradation cannot be completely excluded based solely on this technique. The preservation of the overall chemical structure is a desirable attribute, indicating that the material retains its fundamental chemical identity under the investigated conditions and remains suitable for subsequent reactive modification and processing. Moreover, no new absorption bands indicative of chemical interactions between rPP and LDPE were detected, similar to their virgin counterparts.

### Morphology of Blends via Scanning Electron Microscopy (SEM)

Within the scope of this study, SEM analyses were conducted for compatibilized and uncompatibilized 60/40 PP/LDPE and 60/40 rPP/LDPE samples. An unstable two-phase morphology was observed in both PP/LDPE and rPP/LDPE blends, confirming that PP and LDPE form thermodynamically immiscible polymer blends. The dispersed LDPE phase appeared as spherical particles that were nonuniformly distributed within the PP and rPP matrices. This limited interfacial compatibility may be attributed to the different crystallization behaviors of PP and LDPE, resulting in weak interfacial interactions between the components. The average particle size of the dispersed phase was determined to be 0.38 ± 0.02 μm in the PP/LDPE samples and 0.53 ± 0.03 μm in the rPP/LDPE samples, while spherical LDPE particles larger than 2 μm were also observed in both blends. (Figures 3 and 4). This difference may be related to the relatively higher MFR value of the virgin PP used in this study compared to recycled PP. During melt blending, the higher viscosity of virgin PP may facilitate greater shear-induced breakup of the LDPE phase, leading to a reduction in average particle size.

**Figure 3:**
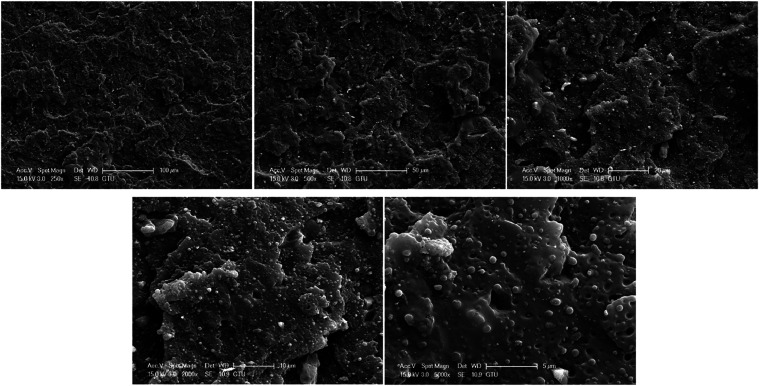
SEM pictures of PP/LDPE blends at different magnifications.

**Figure 4:**
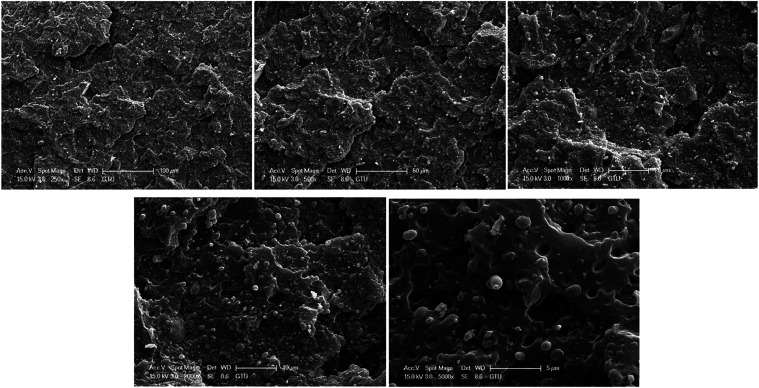
SEM pictures of rPP/LDPE blends at different magnifications.

Figures 5 and 6 present the SEM images of the compatibilized PP/LDPE and rPP/LDPE blends prepared in the presence of the TMPTA coagent. Reactive compatibilization was carried out using peroxide and TMPTA, which promote radical-mediated branching and copolymer formation and cross-linking reactions. The reactive compatibilization process enhanced interfacial interactions between the blend components. The free-radical reactions initiated by peroxides may increase the viscosity of LDPE through chain branching and cross-linking, while simultaneously reducing the viscosity of PP via chain scission^64^ This dual mechanism effectively reduces the viscosity mismatch between the two polymers, thereby improving interfacial compatibility, refining the phase morphology, and enhancing the overall mechanical performance. Moreover, the addition of TMPTA resulted in a highly refined and more homogeneously dispersed multiphase morphology in both systems, indicating improved compatibilization. For instance, the average particle size of dispersed phase was found as 0.13 ± 0.03 μm for rPP7. This suggests that TMPTA enhanced the efficiency of compatibilization. The coagent addition is believed to promote interfacial adhesion by lowering interfacial tension and facilitating closer contact between polymer chains. These modifications may influence the crystallization behavior of the polymers and the molecular alignment at the PP/LDPE bilayer interface, thereby exerting a pronounced impact on interfacial adhesion.

**Figure 5:**
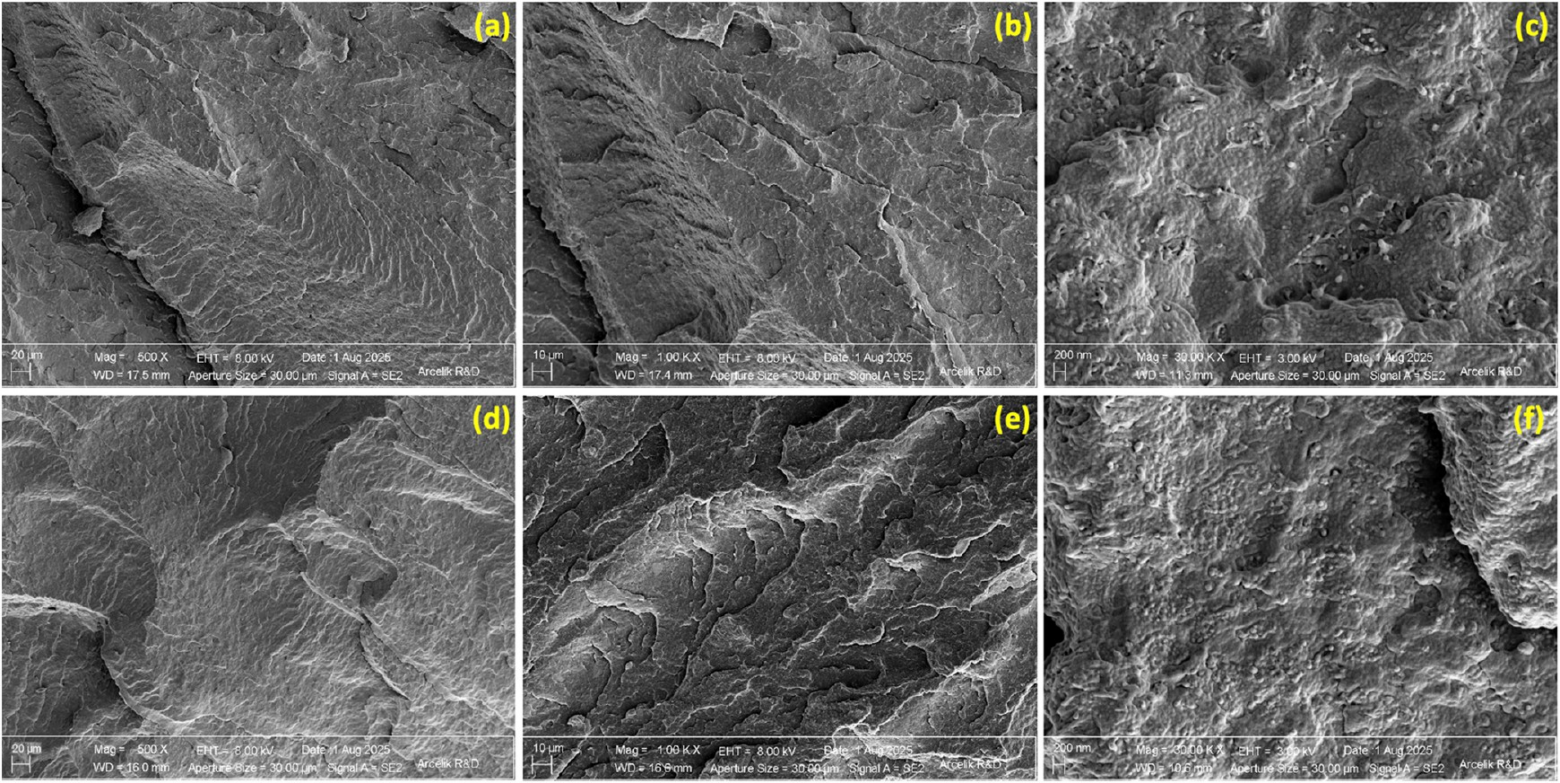
SEM images of prime PP5 (a–c) and PP7 (d–f) at different magnifications.

**Figure 6:**
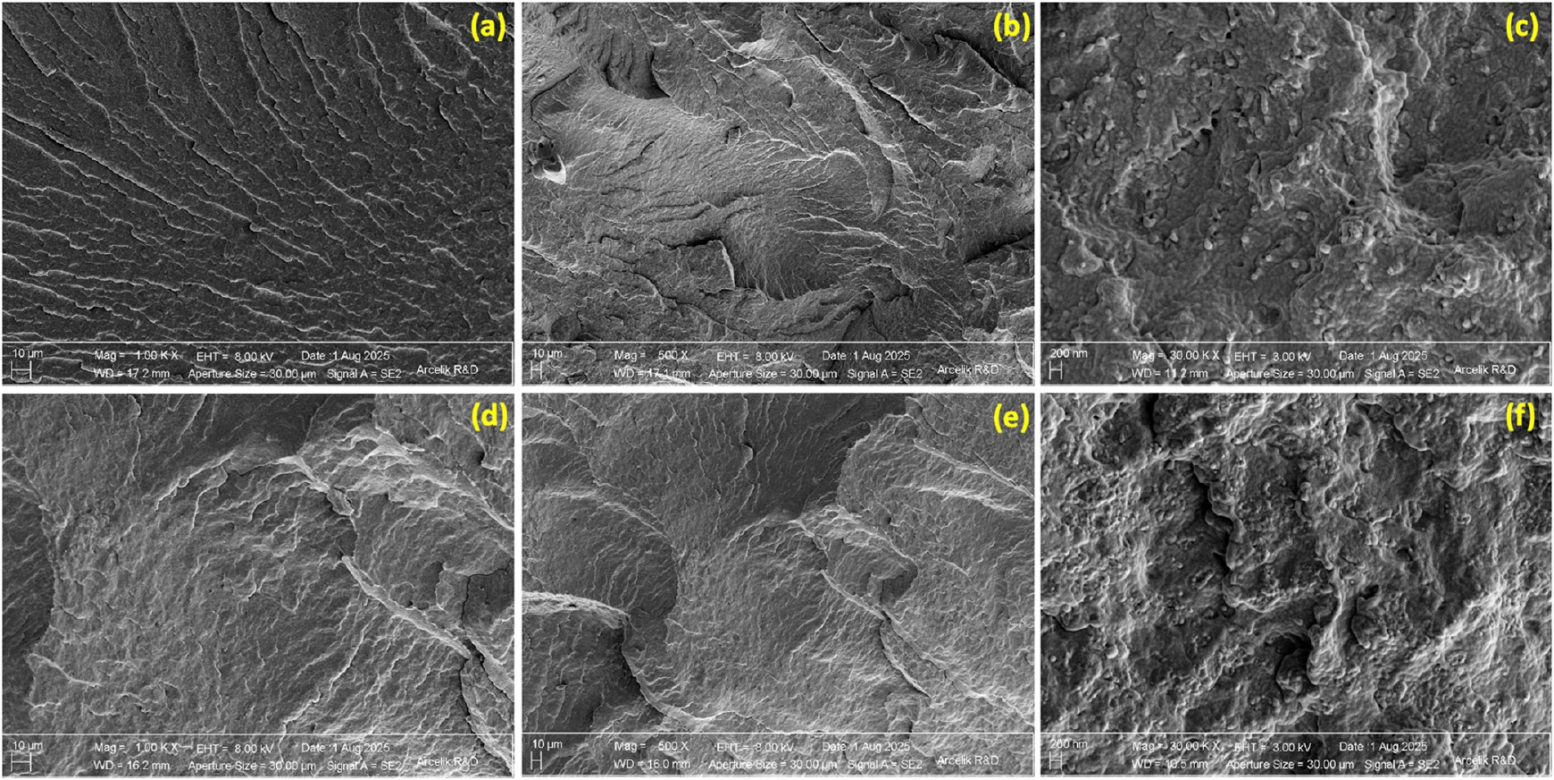
SEM images of rPP5 (a–c) and rPP7 (d–f) at different magnifications.

### Rheological Properties

Figure 7 presents the complex viscosity (η*) of neat homopolymer polypropylene (PP0) and PP containing peroxide, LDPE, and trimethylolpropane triacrylate (TMPTA) as a function of angular frequency. PP0 exhibited the typical shear-thinning behavior of linear isotactic polypropylene. This behavior is attributed to β-scission-induced molecular weight reduction, which decreases zero-shear viscosity and melt elasticity.^65,66^

**Figure 7:**
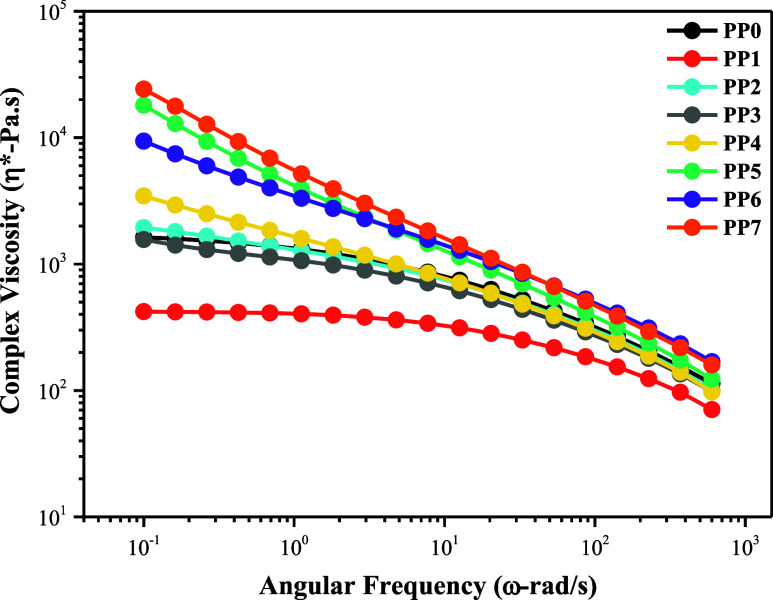
Complex viscosity versus angular frequency of compatibilized and uncompatibilized PP/LDPE blends.

Distinct trends were observed in the blends containing LDPE (PP2 and PP3), depending on the LDPE content. The sample containing 20 wt % LDPE (PP2) exhibited a slightly higher low-frequency viscosity compared to the neat homopolymer PP (PP0), suggesting enhanced elasticity associated with the branched architecture of LDPE. In contrast, the blend containing 40 wt % LDPE (PP3) displayed a complex viscosity curve lying below that of PP0, likely due to the dominance of LDPE’s lower intrinsic viscosity over the structural contributions.^67,68^

Previous studies have reported that the rheological behavior of PP/LDPE blends strongly depends on melt flow rate and molecular architecture, leading to formulation-dependent viscosity–frequency relationships.^53,67^

All PP/LDPE blends compatibilized with TMPTA (PP4, PP5, PP6, and PP7) exhibited pronounced deviations from those without TMPTA. At a 2 wt % TMPTA loading (PP4 and PP5), the samples displayed increased complex viscosity in the low-frequency region and pronounced shear-thinning behavior across the entire frequency range, indicating the formation of long-chain branches resulting from radical recombination reactions. Nam et al. reported a similar phenomenon during the extrusion of PP with TMPTA and peroxide, where TMPTA-containing samples demonstrated altered low-shear viscosities and stronger shear-thinning characteristics.^69^ However, the magnitude of viscosity enhancement observed in that study was smaller than in the present work, likely due to the use of a PP with a much lower MFR. Similarly, Wang et al. found that the incorporation of a polyfunctional monomer, pentaerythritol tetraacrylate, structurally analogous to TMPTA, facilitates the generation of secondary macroradicals, thereby promoting recombination reactions that increase molecular weight through branching, chain extension, and cross-linking.^70^ These trends are consistent with peroxide-coagent-modified polypropylene systems reported in the literature.^69,70^ Increasing the TMPTA content to 5 wt % further amplifies these effects. In particular, PP7 demonstrated the highest viscosity values and a pronounced power law dependence, consistent with extensive long-chain branching and the formation of a structural network reminiscent of high-filler-loaded PP systems, which restricts chain mobility in the low-frequency region.^71,72^ Furthermore, it was observed that LDPE addition enhances the low-frequency viscosity and intensifies the shear-thinning behavior, with the effect becoming more pronounced as the LDPE content increases at constant TMPTA loading.

Figure 8 presents the storage modulus (*G*′) curves of the neat homopolymer PP and its modified counterparts. PP0 exhibited the characteristic behavior of linear isotactic polypropylene, where the storage modulus increased with frequency due to entanglement relaxation. Consistent with the viscosity results, the peroxide-modified PP1 demonstrated the lowest *G*′ values across all frequencies, reflecting reduced elasticity associated with β-scission-induced molecular weight reduction. Wang et al. and Sugimoto et al. attributed the reduction in storage modulus to the decrease in molecular weight, which leads to a lower chain entanglement density, a direct consequence of chain cleavage induced by peroxide degradation.^70,73^ LDPE blended samples (PP2 and PP3) showed an altered storage modulus response. The 20 wt % LDPE blend (PP2) showed an increased storage modulus compared to PP0, indicating enhanced elastic contribution associated with LDPE incorporation. In contrast, the sample containing 40 wt % LDPE (PP3) exhibited lower *G*′ values than PP2, likely due to the dominance of LDPE’s lower intrinsic viscosity over structural elastic contributions. Earlier studies on PP/LDPE blends support this observation, reporting that as LDPE loading increases, the overall elasticity diminishes and the influence of the lower intrinsic viscosity of LDPE becomes more pronounced.^53^ The most significant deviation from PP0 is observed for the TMPTA-compatibilized samples (PP4–PP7) containing peroxide. The incorporation of TMPTA introduces long-chain branches that generate slower relaxation modes, manifested as substantially higher *G*′ values, particularly at low frequencies. The magnitude of this deviation decreased with increasing frequency, as the influence of the lower intrinsic viscosity of the system became dominant.

**Figure 8:**
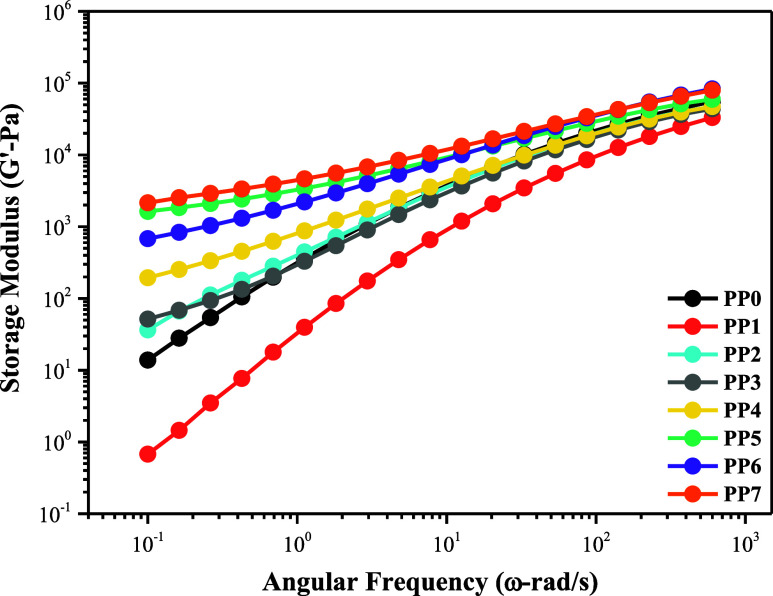
Storage modulus versus angular frequency of compatibilized and uncompatibilized PP/LDPE blends.

Figure 9 illustrates the loss modulus (*G*″) curves of the individual samples. The smooth increase in *G*″ with frequency observed for neat isotactic polypropylene (PP0) is characteristic of the terminal flow region of linear PP melts. In the peroxide-modified sample (PP1), *G*″ values are consistently lower than those of PP0 across all frequencies, reflecting molecular weight reduction caused by chain scission. The resulting decrease in molecular size constrains viscous dissipation, leading to diminished *G*″ values.

**Figure 9:**
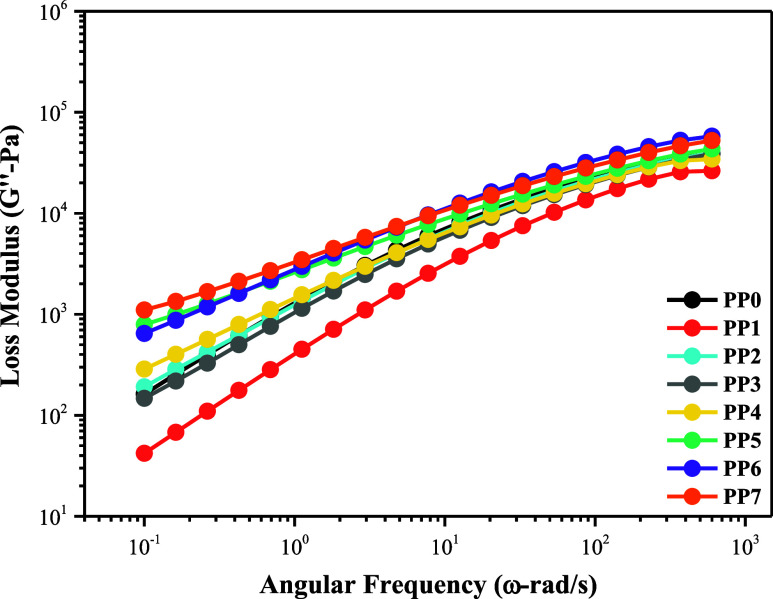
Loss modulus versus angular frequency of compatibilized and uncompatibilized PP/LDPE blends.

Samples incorporating LDPE exhibited trends similar to PP0, indicating that LDPE addition has a limited influence on overall viscous dissipation compared to its more pronounced effect on elasticity. However, minor variations are evident in the low-frequency region for the 20 wt % LDPE blend (PP2); an increase in *G*″ was observed, suggesting an enhanced viscous response associated with structural modification, whereas for the 40 wt % LDPE blend (PP3), the lower intrinsic viscosity of LDPE predominates, resulting in decreased *G*″ values. In contrast, samples compatibilized with both TMPTA and peroxide exhibit markedly different behavior compared to those containing only LDPE. At 2 wt % TMPTA loading (PP4 and PP5), a substantial increase in *G*″ relative to PP0 and PP2 was observed, particularly at lower frequencies, indicating modification of the molecular architecture through long-chain branching and possible intermolecular coupling. This behavior is primarily attributed to the broadened molecular weight distribution generated through peroxide–coagent reactions. The effect becomes even more pronounced at 5 wt % TMPTA (PP6 and PP7), suggesting the presence of slower relaxation modes and a broader relaxation spectrum associated with long-chain branching and limited cross-linking at higher coagent concentrations. Among all samples, PP7 exhibited the highest *G*″ values, demonstrating the synergistic influence of elevated LDPE content and TMPTA concentration on the development of a highly constrained viscoelastic structure. Similar increases in *G*″ and flattening of terminal slopes have been reported for long-chain-branched polypropylene produced by reactive extrusion^74,52^ and for PP blends containing high melt strength PP.^75^

Cole–Cole plots provide useful insight into relaxation behavior in polymer melts.^74^ Structural variations among the samples are further elucidated in the Cole–Cole plots presented in Figure 10. The neat homopolymer PP0 exhibited a nearly semicircular curve, characteristic of linear isotactic polypropylene with a narrow relaxation spectrum. In contrast, the pronounced reduction in the arc radius for the peroxide-modified sample PP1 reflects a decrease in molecular weight due to chain scission initiated by peroxide-induced degradation. The PP2 sample displayed an enlarged curve, suggesting the presence of slower relaxation dynamics associated with LDPE incorporation. PP3 exhibited a broader and less regular arc, indicating a more complex relaxation behavior influenced by the lower intrinsic viscosity of LDPE. A strong deviation from the semicircular profile is observed for the TMPTA-compatibilized samples (PP4-PP7), where pronounced upturned tails appear at high viscosities, indicative of broadened relaxation spectra and extended relaxation modes associated with long-chain branching and possible intermolecular cross-linking at higher TMPTA loadings. This deviation serves as a characteristic rheological signature commonly reported for long-chain branched and partially cross-linked high-melt-strength polypropylene systems.

**Figure 10:**
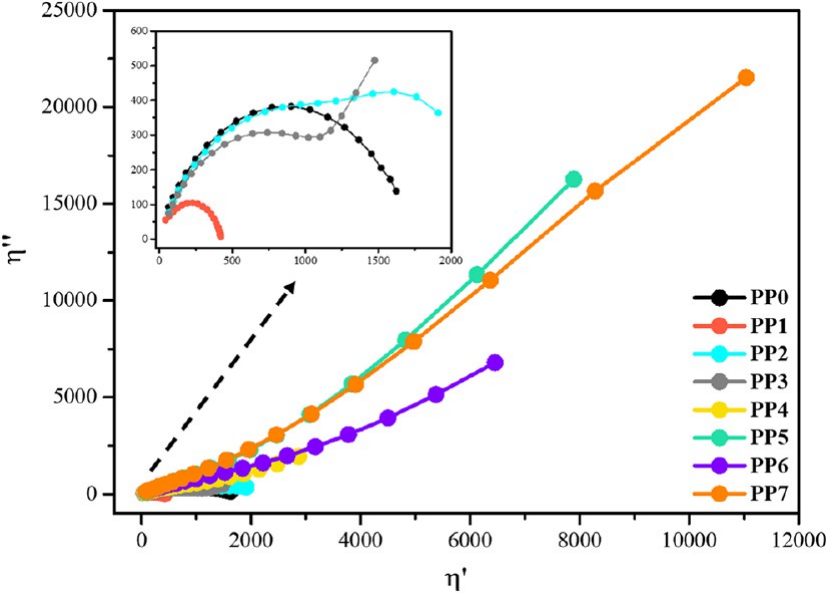
Cole–Cole plots of compatibilized and uncompatibilized PP/LDPE blends.

Figure 11 compares the complex viscosity (η*) values of both recycled and virgin polypropylene materials and their corresponding modified formulations. The recycled homopolymer (rPP0) exhibited a lower molecular weight compared to the virgin PP0, as evidenced by its lower viscosity and broader Newtonian plateau. This behavior is typical of recycled polymers and is primarily attributed to chain scission, which occurs during repeated thermal processing cycles. The modified formulations based on both virgin and recycled PP demonstrate comparable rheological behavior under TMPTA and peroxide modification. All samples display markedly increased low-frequency viscosities accompanied by diminished Newtonian regions, indicative of modified molecular architecture involving long-chain branching and possible intermolecular cross-linking. Interestingly, the recycled-based formulations exhibit even higher viscosity values than their virgin counterparts. This observation is reasonable, as recycled grades often contain oxidized and partially degraded chains that are more susceptible to radical generation during reactive processing. The presence of excess radicals likely promotes additional intermolecular reactions, potentially increasing the extent of cross-linking and a broader relaxation spectrum, particularly in the low-shear region, surpassing those observed in PP5 and PP7. The presence of possible cross-linked structures was evaluated through gel content measurements. While rPP7 exhibited 11% gel content, this value was determined to be 6% for the PP7 sample. The relatively higher cross-linking density led to increased complex viscosity in both TPMTA-compatibilized blends. Moreover, the lower cross-linking density resulted in improved reprocessability.

**Figure 11:**
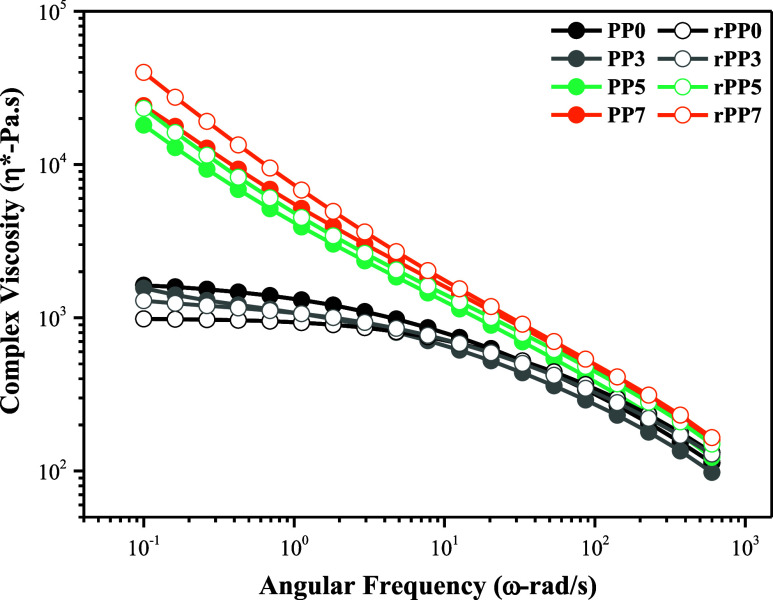
Complex viscosity versus angular frequency of compatibilized and uncompatibilized PP/LDPE and rPP/LDPE blends.

Figure 12 shows Cole–Cole plots comparing virgin and recycled grades. The observed trends are consistent with the complex viscosity results. rPP0 has a reduced semicircular profile, reflecting its lower molecular weight compared to PP0. rPP5 and rPP7 exhibit more pronounced upturns at higher viscosity values in the low-frequency region, indicating broader relaxation spectra compared to PP5 and PP7. These observations suggest that reactive modification alters the relaxation behavior of recycled systems, leading to enhanced low-frequency viscosity and extended relaxation modes associated with long-chain branching and possible intermolecular cross-linking at higher coagent concentrations.

**Figure 12:**
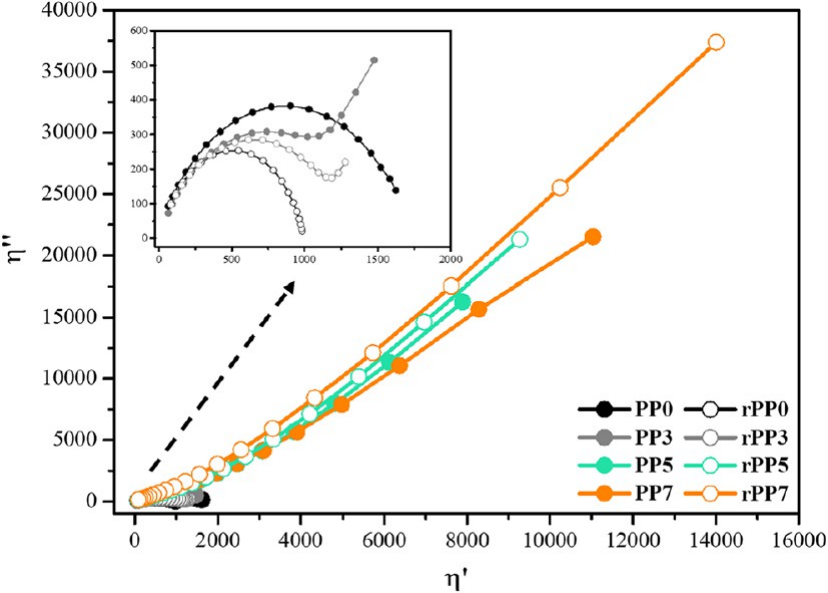
Cole–Cole plots of compatibilized and uncompatibilized PP/LDPE and rPP/LDPE blends.

Figures 13 and 14 present damping factor (tan δ = *G*″/*G*′) as a function of frequency for all samples studied. Tan δ provides information regarding balancing between viscous dissipation and elastic storage, where high tan δ (>1) reflects viscous-dominated behavior, low tan δ (<1) reflects elastic-dominated behavior, and flat curves are associated with broadened relaxation spectra.

**Figure 13:**
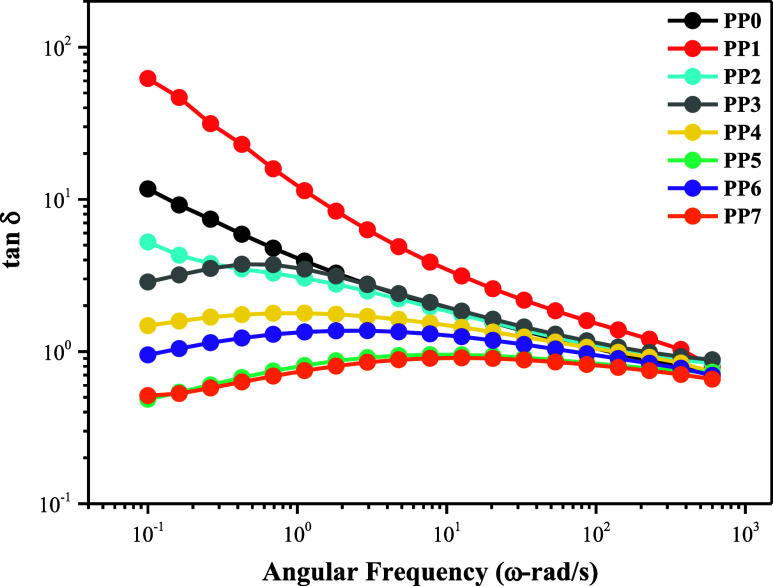
Tan δ versus angular frequency of compatibilized and uncompatibilized PP/LDPE blends.

**Figure 14:**
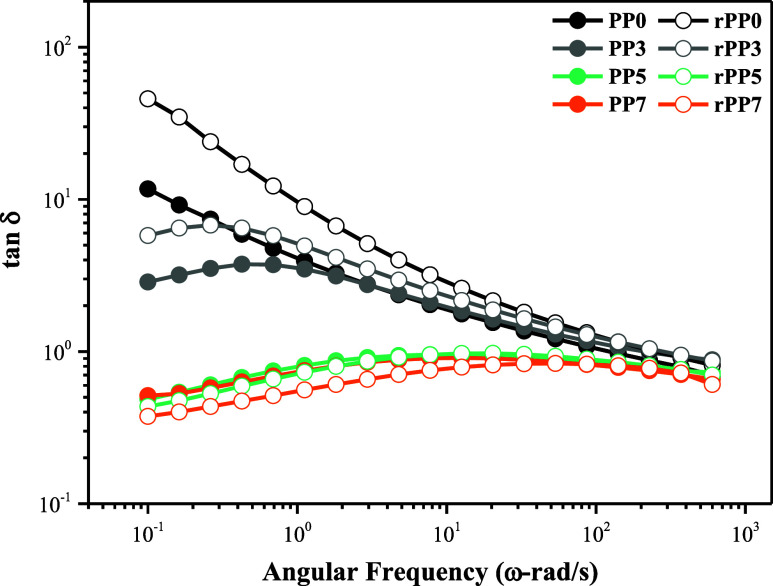
Tan δ versus angular frequency of compatibilized and uncompatibilized rPP/LDPE blends.

Consistent with the trends observed in the viscosity and modulus data, the tan δ in Figure 13 illustrates the relative dominance of viscous and elastic contributions in each sample. The neat homopolymer PP0 exhibited tan δ values well above unity at low frequencies, which gradually decreased with increasing frequency, indicating a viscous-dominated terminal region while maintaining the characteristic viscoelastic balance of linear isotactic polypropylene. This behavior aligns with its moderately complex viscosity and the intermediate crossover point observed in the modulus curves.^74^

The peroxide-modified PP1 exhibited elevated tan δ values at low frequencies, which directly corresponds to strong viscous dissipation dominating over elasticity. This result aligns with reduced low-complex viscosity and storage modulus and a crossover point shifted to higher frequency.^74,76^ A relative increase in elasticity is evident in the LDPE-blended samples (PP2 and PP3), which exhibit lower tan δ values than PP0 across most of the frequency range. The inherent long-chain branching of LDPE introduces slow relaxation modes that enhance the elastic response; however, in the case of PP3, this effect is partially offset by the predominance of the lower intrinsic viscosity of the LDPE phase.^75,77^

The prominent reduction in tan δ has been observed in TMPTA-loaded samples (PP4–PP7, rPP5, and rPP7), where tan δ values stay under 1 across a broad range of frequencies. These results indicate that elasticity strongly dominates, which directly matches with high complex viscosity at low frequencies and the elevated *G*′ and *G*″ values. This type of behavior is amply documented for long-chain branched polypropylene synthesized by peroxide–coagent reactive extrusion, where extended relaxation spectra and sluggish modes lead to enhanced melt elasticity.^69,71,74^ The effect was most pronounced for PP7 and rPP7, where the combination of elevated LDPE content and 5 wt % TMPTA resulted in the lowest tan δ values and highest low-frequency viscoelastic response, suggesting the development of a highly constrained molecular structure involving long-chain branching and possible intermolecular cross-linking.^69,78,79^

Despite the pronounced increase in low-frequency viscosity and modulus values, no abrupt upturns, discontinuities, or processing instabilities indicative of macroscopic gel formation were observed during extrusion or injection molding.

The reduced tan δ values and elevated low-frequency viscosity observed for TMPTA-modified formulations enhance melt elasticity under extensional flow conditions encountered during injection molding foaming. This improved viscoelastic response increases resistance to cell coalescence and collapse, thereby promoting more stable cellular structures.

### Mechanical Properties

Tensile and Izod impact tests were conducted to assess the mechanical performance of prime and recycled polypropylene, peroxide-modified PP, and compatibilized PP/LDPE and rPP/LDPE blends. Figure 15 presents the variations in mechanical properties observed across these different formulations.

**Figure 15:**
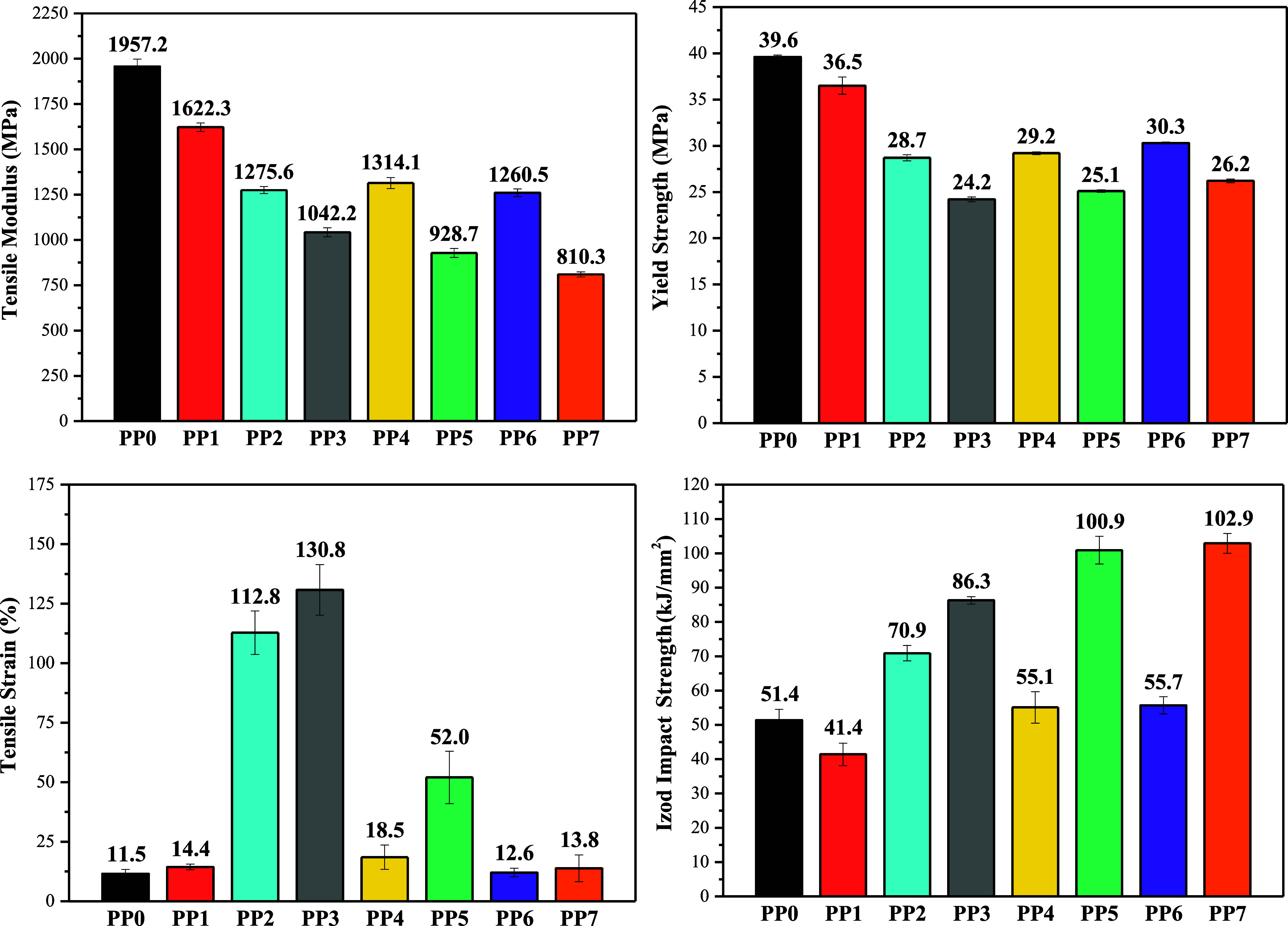
Changes in mechanical properties of PP and compatibilized and uncompatibilized PP/LDPE blends.

Neat polypropylene (PP0) displayed the highest tensile modulus (1957 MPa) and yield strength (39.6 MPa), attributable to its semicrystalline and rigid structure. In PP1, the yield strength decreased to 36.5 MPa, reflecting the reduction in effective molecular weight caused by DHBP-induced chain scission. Incorporation of LDPE at 20–40 wt % (PP2, PP3) led to a marked decrease in tensile modulus and yield strength. SEM analysis revealed that this trend arises from the low miscibility of PP and LDPE, as evidenced by distinct LDPE domains with weak interfacial adhesion. The inefficient stress transfer across the interface diminishes mechanical reinforcement.^13^ However, the ductile LDPE domains functioned as localized stress concentrators, deforming plastically under load and thereby significantly increasing elongation at break (112.8% in PP2 and 130.8% in PP3).^80^ PP/LDPE blends compatibilized with TMPTA (PP4-PP7) exhibited substantially altered morphology as mentioned in SEM analyses. SEM images revealed finer LDPE domains and rougher fracture surfaces, indicating that long-chain branching and partial cross-linking improved compatibility between PP and LDPE. Consequently, PP4 displayed the highest tensile modulus among the compatibilized blends, consistent with more effective stress transfer through enhanced interfacial interactions. Conversely, elongation at break decreased relative to PP2 and PP3, as the branched structure restricted chain mobility and reduced the capacity for plastic deformation.^29^

Impact strength results further emphasize the critical role of morphology. In PP2 and PP3, coarse LDPE domains enhanced toughness by absorbing impact energy, as corroborated by SEM observations of ductile fracture surfaces.^80^ The incorporation of TMPTA and peroxide produced even higher impact strength in PP5 and PP7 due to a synergistic effect: SEM analysis showed smaller, more uniformly distributed LDPE domains that effectively hindered crack propagation, while the branched polypropylene network provided additional free volume for energy dissipation. In contrast, lower LDPE content in PP4 and PP6 was less effective in arresting crack growth, resulting in only modest improvements in impact strength.

Overall, the mechanical performance of PP/LDPE blends is strongly correlated with their morphology. SEM evidence confirms that weak interfacial interaction accounts for the reduced modulus and yield strength, whereas finer LDPE domains and enhanced interfacial interactions in branched systems are responsible for improved impact resistance.^13^ The ratio of TMPTA to peroxide in the PP/LDPE blends, therefore, governs the balance between stiffness, ductility, and toughness.

Recycled neat polypropylene (rPP0) exhibited the highest tensile modulus and yield strength among the recycled samples due to its semicrystalline structure; however, it displayed limited elongation and relatively low impact strength (Figure 16). It can be attributed to the chain scission and impurities arising from the recycling process that promoted brittle fracture surfaces and restricted toughness.^11,81,82^ rPP/LDPE blend including 40 wt % LDPE (rPP3) showed higher elongation at break due to the higher elasticity of virgin LDPE. On the other hand, the rPP/LDPE blend exhibited lower tensile modulus and yield strength due to poor interfacial interaction between the components.

**Figure 16:**
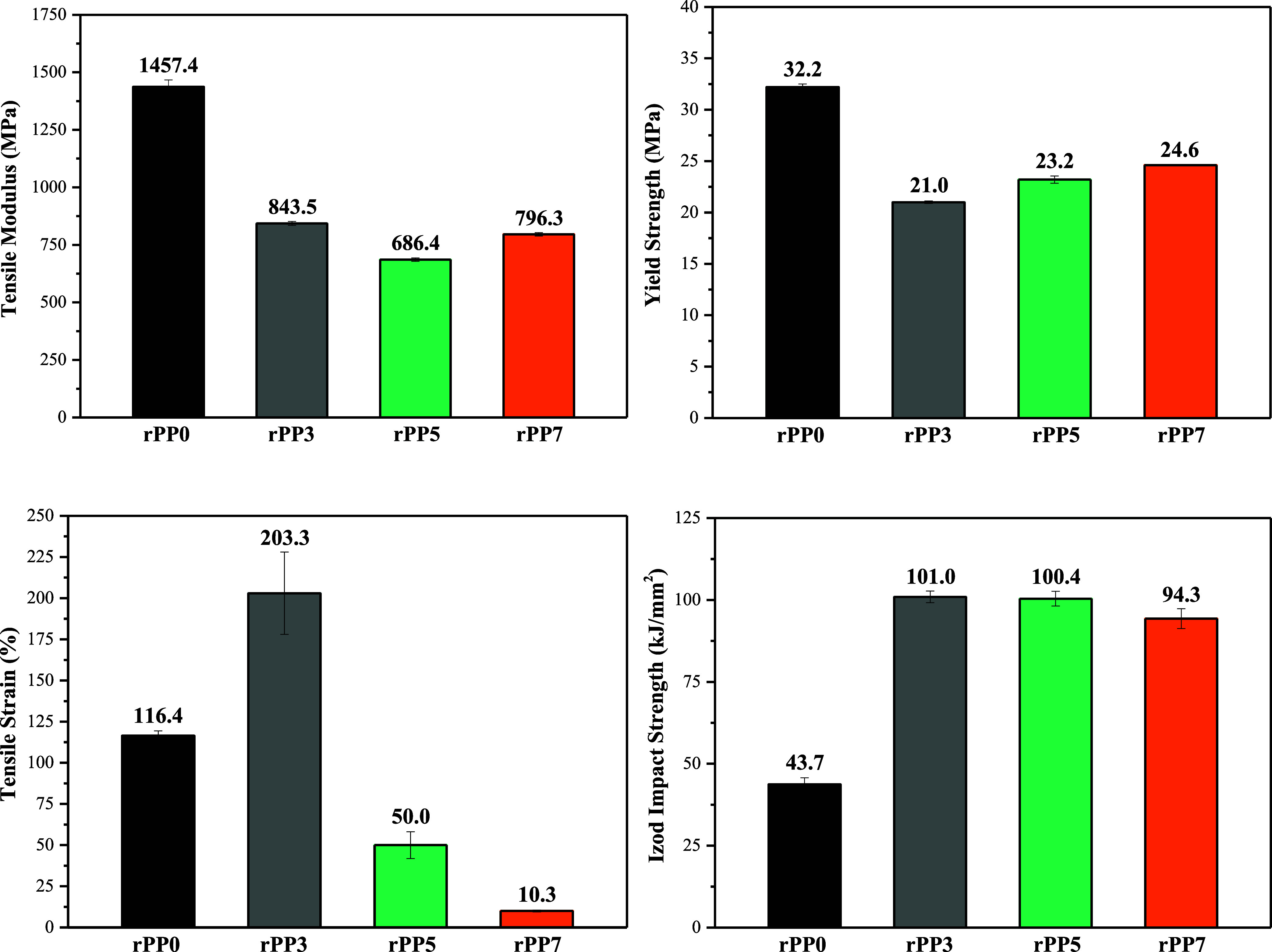
Changes in mechanical properties of rPP and compatibilized and uncompatibilized rPP/LDPE blends.

The incorporation of LDPE alongside TMPTA and peroxide in rPP5 markedly altered both morphology and mechanical performance. Although the tensile modulus, yield strength, and tensile strain decreased significantly, moderate long-chain branching induced by TMPTA/DHBP contributed to improved toughness.^83^ The ductile nature of LDPE and its capacity to absorb impact energy dominated the fracture mechanism, enhancing toughness despite the reduction in stiffness.^13^ In rPP7, increasing the TMPTA content in the presence of peroxide produced finer and more homogeneously dispersed LDPE domains compared to rPP5, as evidenced by SEM. The rougher fracture surfaces reflected the formation of long-chain branching and partial cross-linking, which restricted chain mobility.^29^ Mechanically, this manifested as a slight recovery in modulus and yield strength relative to rPP5, while elongation at break decreased sharply to 10%, indicating the onset of more brittle behavior. Despite the reduction in ductility, high impact strength was maintained, likely due to the refined LDPE domain structure, which effectively deflects crack propagation and facilitates energy dissipation. In summary, rPP0 is stiff but relatively brittle; rPP5 exhibits enhanced toughness due to smaller LDPE domains and long-chain branching; and rPP7 is tougher, reflecting a balance between domain refinement. SEM observations strongly corroborate the mechanical data, highlighting that the morphology and dispersion of LDPE critically govern the balance between stiffness, ductility, and toughness. It should be emphasized that the observed balance among stiffness, ductility, and impact resistance is formulation-dependent and reflects the specific LDPE content, TMPTA concentration, and reactive processing conditions examined in this study, rather than representing a universally optimal composition.

### DSC Results

Differential scanning calorimetry (DSC) was employed to examine the thermal transitions of pure PP, pure LDPE, compatibilized and uncompatibilized PP/LDPE, and rPP/LDPE blends. The thermograms corresponding to the cooling and second heating cycles are presented in Figures 17A,B and 18A,B, respectively. A summary of the thermal transition data is provided in Table 2.

**Figure 17:**
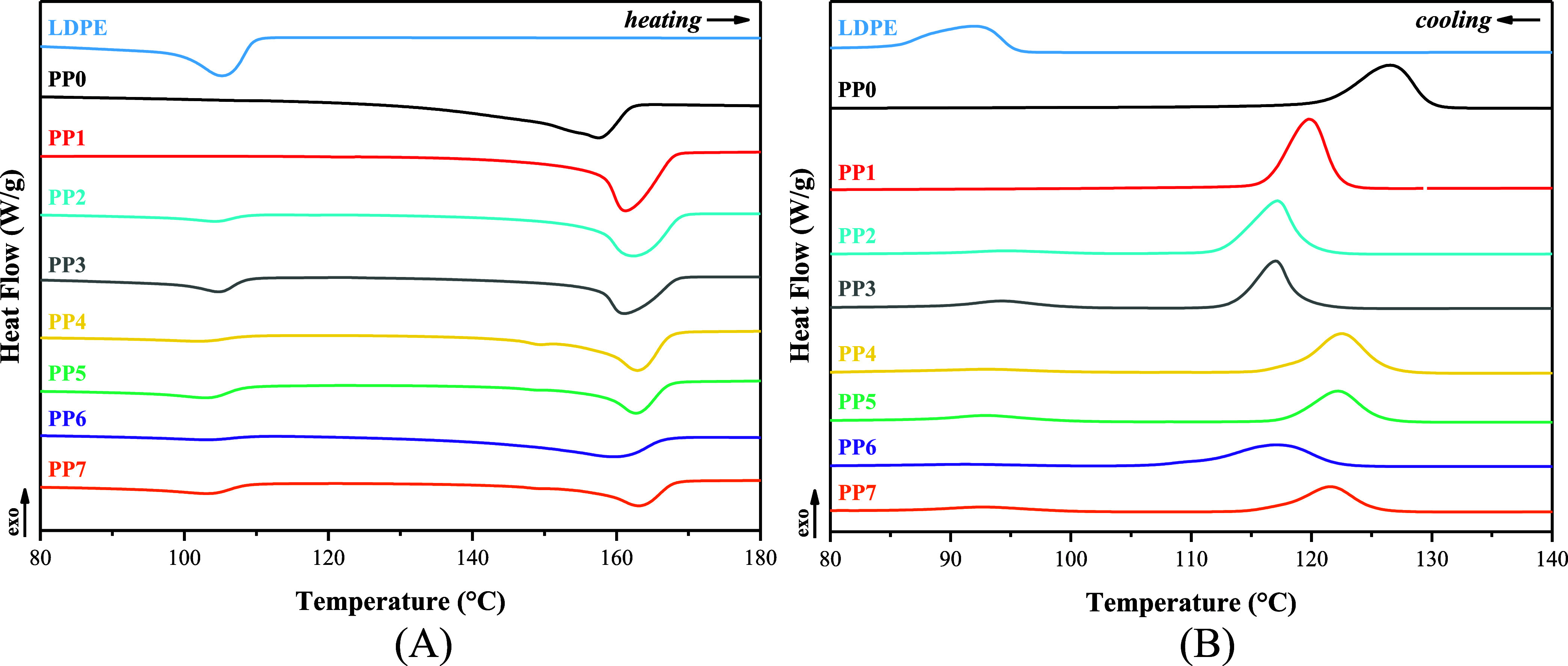
(A) Second heating and (B) cooling DSC thermograms of LDPE, PP and compatibilized and uncompatibilized PP/LDPE blends.

**Figure 18:**
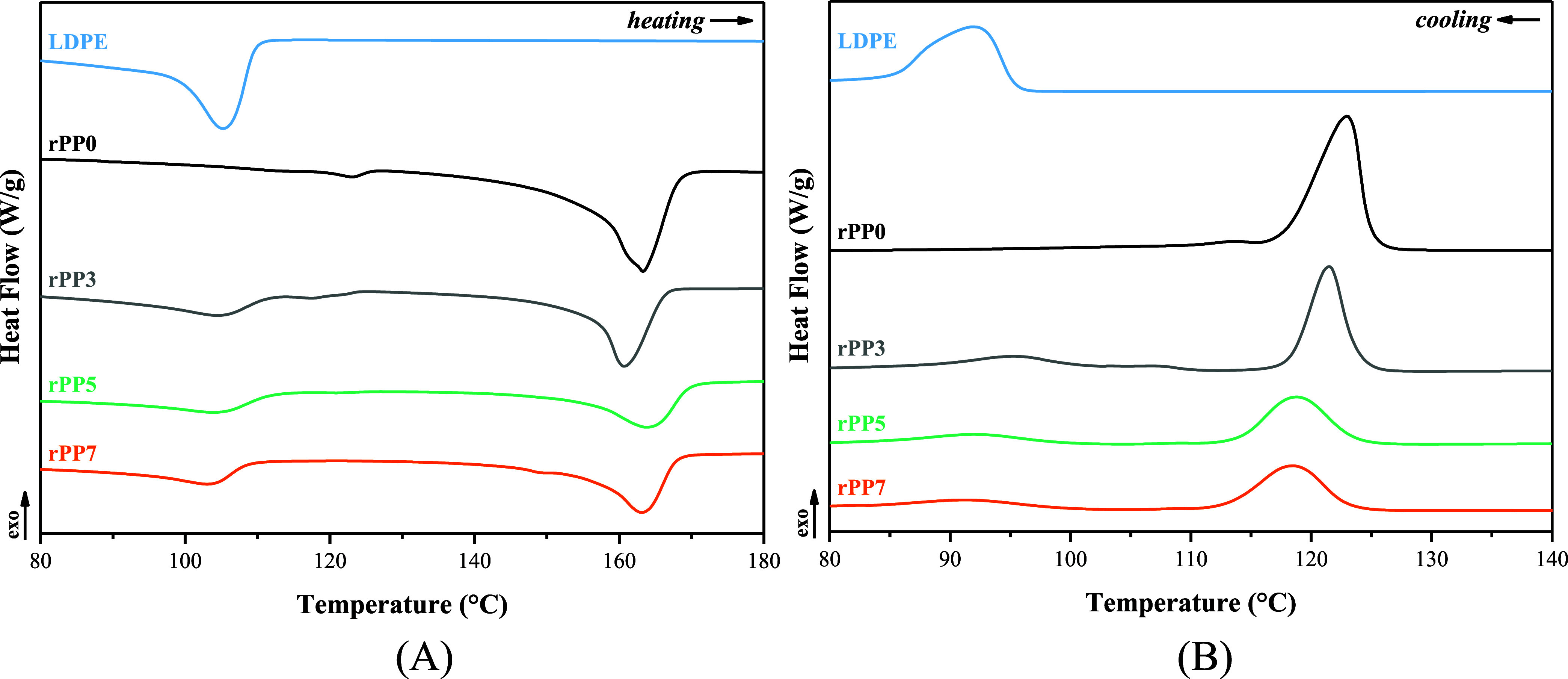
(A) Second heating and (B) cooling DSC thermograms of LDPE, rPP and compatibilized and uncompatibilized rPP/LDPE blends.

**Table 2:** Thermal Properties of Neat Polymers, Compatibilized and Uncompatibilized PP/LDPE and rPP/LDPE Blends

sample	*T*_m_ (°C) LDPE	*T*_c,onset_ (°C) LDPE	Δ*H*_m_ (J/g)LDPE	Δ*H*_c_ (J/g) LDPE	*T*_m_ (°C) PP	*T*_c,onset_ (°C) PP	Δ*H*_m_ (J/g) PP	Δ*H*_c_ (J/g) PP	Xc,PP (%)	%Xc,LDPE (%)
**LDPE**	105.1	95.5	110.3	79.2	-	-	-	-	-	38.3
**PP0**	-	-	-	-	157.4	129.9	100.7	102.6	48.6	-
**PP1**	-	-	-	-	161.2	122.6	95.6	104.6	46.4	-
**PP2**	103.9	101.1	10.7	8.7	161.5	120.4	71.3	71.6	43.0	18.6
**PP3**	94.7	100.5	29.7	23.6	161.4	120.7	52.4	50.7	42.2	25.8
**PP4**	101.9	101.3	16.8	21.3	162.8	126.4	78.4	76.0	48.8	29.0
**PP5**	102.9	99.9	40.6	43.2	162.6	125.9	53.6	54.9	44.8	35.2
**PP6**	102.8	99.9	11.4	10.2	159.4	122.3	64.1	63.4	41.4	19.7
**PP7**	102.9	100.5	33.9	38.1	163.0	125.4	50.9	50.0	45.0	29.4
**rPP0**	-	-	-	-	163.2	124.9	121.1	112.9	58.5	-
**rPP3**	104.5	101.0	33.1	24.8	160.3	122.6	42.8	43.8	34.5	28.7
**rPP5**	104.3	99.8	27.2	18.1	163.8	123.6	42.9	42.0	35.8	23.6
**rPP7**	103.9	99.9	34.5	28.5	163.8	123.1	42.4	42.2	35.5	29.9

Neat LDPE exhibited a melting temperature (*T*_m_) of 105.1 °C, while pure PP (PP0) displayed a melting point of 157.4 °C. It was observed that the melting temperature of PP shifts to a relatively higher value with the addition of peroxide, while the melting enthalpy decreased from 100.7 to 95.6 J/g. This behavior is attributed to the formation of less-ordered crystals in the PP phase as a result of chain scission reactions occurring in the presence of peroxide. Moreover, the crystallization onset temperature (*T*_c,onset_) of the pure PP was markedly affected by the incorporation of peroxides. In the pure PP (PP0), *T*_c,onset_ was 129.9 °C. The introduction of peroxide alone (PP1) led to a pronounced reduction in *T*_c,onset_ to 122.6 °C, a characteristic indicator of chain scission-induced degradation, which diminishes molecular weight and impedes nucleation.^47,84^ The blends (PP2 and PP3) consistently exhibited dual melting and crystallization peaks, indicating that PP and LDPE crystallize and melt independently, consistent with the presence of immiscible phases. The invariance of the melting temperatures across different blend compositions further supports the absence of substantial molecular-level mixing between the two polymers.^47,84^ In PP/LDPE blends, the melt crystallization onset temperature of PP occurred at a temperature approximately 9 °C lower, whereas the melt crystallization temperature of LDPE shifted to higher values. This behavior indicated that the crystallization of the PP phase is suppressed by LDPE, while the PP phase acted as a nucleating agent for LDPE. In addition, both components in the PP/LDPE blend exhibit a significant decrease in their Δ*H*_c_ values compared to their neat counterparts. This trend was consistent with the observed reduction in percent crystallinity. These findings suggest that the spherulite density of both PP and LDPE decreases within the LDPE/PP blend. Similar findings were also observed when 40% LDPE was incorporated into rPP. For instance, the Δ*H*_c_ value of rPP decreased from 112.9 to 43.8 J/g (Table 2).

A pronounced reversal of the previously observed trend occurred upon the concurrent addition of TMPTA. In samples PP4 and PP5, which contained 2 wt % TMPTA, the melt crystallization onset temperature of the PP phase increased markedly to 126.4 and 125.9 °C as a comparison with uncompatibilized PP/LDPE blends, respectively. This behavior indicated that TMPTA functions as a branching agent, promoting the formation of long-chain branches along the PP backbone that serve as efficient nucleation sites.^85,86^ Moreover, TMPTA can react with PP macroradicals, thereby suppressing β-scission reactions.^71^ At higher TMPTA concentrations, this advantage diminished for PP, including 20 wt % LDPE (PP6), with *T*_c,onset_ value of PP decreasing to 122.3 °C. This decline suggested that an excessively high branch density restricts the chain mobility required for efficient crystallization.^87^ When evaluating the melting temperatures of the TMPTA-containing samples, it was observed that the melting temperature of the PP phase remained unchanged. However, in the 60/40 LDPE/PP blend, the melting temperature of the LDPE phase increased by approximately 9 °C, regardless of the TMPTA content. The shift of the LDPE melting temperature toward that of PP is attributed to enhanced interfacial interactions between the two components. Regardless of TMPTA concentration, following reactive compatibilization, the Δ*H*_c_ values of the LDPE phase increased in both 80/20 and 60/40 PP/LDPE blends. In contrast, for the PP phase, the Δ*H*_c_ value increased with the addition of 2% TMPTA; however, it decreased at 5% TMPTA. These results reveal a delicate balance between branching and cross-linking, wherein partial cross-linking ultimately hampers crystallization. After reactive compatibilization, no significant changes were observed in the thermal properties of the PP and LDPE phases within the PP/LDPE blends. However, the recycled homopolymer (rPP0) showed an initial crystallinity of 58.5%, which exceeded that of its virgin counterpart, which is commonly the effect of service-life-induced chain scission; it forms shorter mobile chains favoring crystallization.^87^ The blending of rPP with LDPE (rPP5) resulted in the comprehensive loss of polypropylene crystallinity such that it fell to 35.8%, which signifies a much higher decline compared to that observed in the virgin counterpart blend. This implies that the shorter chains within the rPP favor easier disruption.

### Structure of the Foams

Figure 19 shows the structure of foams of PP0, PP5, and PP7 samples. Rheology analyses revealed that samples PP5 and PP7 exhibited pronounced branching and/or cross-linking structures. The reference sample (PP0) displayed the lowest complex viscosity (η*) at low frequencies, characteristic of linear isotactic polypropylene with limited melt strength. In contrast, PP5 demonstrated significantly higher η* and pronounced shear-thinning behavior, indicative of long-chain branching and strain-hardening effects. PP7 exhibited the highest low-frequency η*, confirming the presence of extensive branching and cross-linking, typical of high melt strength PP foams. The observed differences in foam morphology can be directly correlated with the rheological behavior of the corresponding melts. Formulations exhibiting increased low-frequency complex viscosity, elevated storage modulus, and reduced tan δ values demonstrated enhanced melt elasticity and strain-hardening behavior. These rheological characteristics improved melt strength under the extensional flow conditions encountered during injection molding, thereby increasing resistance to bubble coalescence and cell collapse. The foaming results were consistent with these rheological observations. PP0 exhibited the lowest cell density (6.6 × 10^7^ cells/cm^3^) and the largest mean cell size (≈9.4 μm), reflecting its weak melt strength, which promotes cell coalescence and poor structural stabilization. Conversely, PP5 showed the highest cell density (1.1 × 10^8^ cells/cm^3^) and the smallest average cell diameter (≈7.5 μm). The pronounced strain-hardening behavior of PP5 enhanced melt stability during mold filling, stabilizing cell walls and effectively suppressing bubble collapse, leading to refined microcellular structures.^88^ Despite its high viscosity, PP7 exhibited a lower cell nucleation density (*N*_0_ = 8.7 × 10^7^ cells/cm^3^) compared to PP5, suggesting that excessive branching and partial cross-linking may hinder nucleation efficiency, resulting in fewer but slightly larger cells (≈7.8 μm) (Table 3). Excessively high melt elasticity may restrict bubble nucleation kinetics and limit gas diffusion during cell growth, thereby reducing the number of effective nucleation sites despite improved resistance to coalescence. This observation aligns with previous findings indicating that overly high branching density can suppress nucleation even when coalescence is minimized.

**Figure 19:**
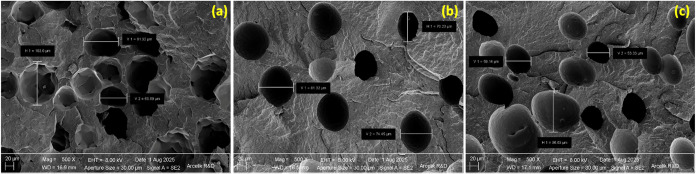
SEM image of chemically foamed (a) Prime PP0, (b) PP5, and (c) PP7.

**Table 3:** Average Cell Sizes and Cell Density of PP, PP/LDPE, and rPP, rPP/LDPE Polymer Composite Foams

sample	cell count, *n* (avg)	average cell diameter, *D*_0_ (μm)	median diameter (μm)	cell density, *N*_0_ (cells/cm^3^)
**PP0**	372 ± 21	9.36 ± 0.33	6.25 ± 0.34	(6.59 ± 0.54) × 10^7^
**PP5**	520 ± 29	7.48 ± 0.22	5.34 ± 0.02	(1.10 ± 0.09) × 10^8^
**PP7**	441 ± 48	7.80 ± 0.50	5.48 ± 0.13	(8.72 ± 1.43) × 10^7^
**rPP0**	418 ± 40	9.19 ± 0.73	5.90 ± 0.22	(7.58 ± 1.07) × 10^7^
**rPP5**	524 ± 104	7.77 ± 0.46	5.49 ± 0.02	(1.12 ± 0.33) × 10^8^
**rPP7**	375 ± 12	8.32 ± 1.31	5.45 ± 0.32	(6.65 ± 0.32) × 10^7^

SEM images revealed distinct differences in cell morphology among rPP0, rPP5, and rPP7 (Figure 20). Specifically, rPP0 exhibited relatively large cells (mean diameter ≈ 9.2 μm; median ≈ 5.9 μm) with a cell density of approximately 7.6 × 10^7^ cells/cm^3^, indicative of reduced melt strength and limited nucleation accompanied by extensive cell coalescence.^89^ In contrast, rPP5 displayed the most refined cell morphology, characterized by the smallest mean cell diameter (∼7.8 μm) and the highest cell density (1.1 × 10^8^ cells/cm^3^). This improvement can be attributed to enhanced melt elasticity resulting from the synergistic effects of LDPE incorporation and chain branching induced by TMPTA, which together promote nucleation.^88^ rPP7 exhibited an intermediate morphology, with a mean cell diameter of approximately 8.3 μm and a reduced cell density of around 6.7 × 10^7^ cells/cm^3^. These findings suggest that higher cross-linking and overbranching suppress nucleation efficiency and impede proper cell stabilization.^58^ Overall, these observations are consistent with previous studies on high melt strength and long-chain branched polypropylene foams, confirming that an optimal degree of branching maximizes cell density and promotes uniform foam morphology.^90^

**Figure 20:**
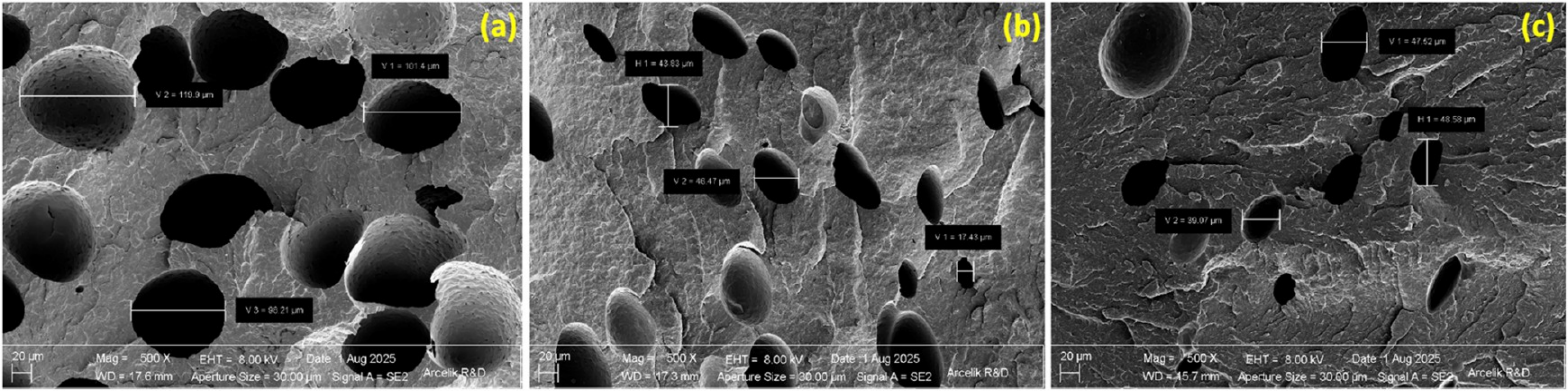
SEM image of chemically foamed (a) rPP0, (b) rPP5, and (c) rPP7.

Overall, the rheological characteristics of the TMPTA-modified systems, particularly reduced tan δ values and enhanced low-frequency viscoelastic response, improve melt stability under the extensional flow conditions of injection molding, thereby limiting bubble coalescence and collapse and promoting finer and more uniform cellular morphologies.

### Mechanical Properties of Foamed Materials

Following the foaming process, the material density decreased by approximately 5–20%, confirming the successful formation of a cellular morphology. For instance, the density of PP1 decreased from 0.91 to 0.74 g/cm^3^, which is consistent with the volumetric expansion induced by the decomposition of the foaming agent. This reduction in density directly affects the mechanical performance of the material, as the increased void fraction and reduced effective load-bearing cross-sectional area led to a decrease in stiffness and strength. After foaming, all samples exhibited a pronounced reduction in both tensile modulus and yield strength. Specifically, for PP5, the modulus decreased from 928 to 717 MPa and the yield strength from 25.1 to 18.0 MPa, whereas for PP7, the modulus dropped from 810 to 701 MPa and the yield strength from 26.2 to 17.5 MPa (Figure 21). Among the examined formulations, PP7 showed the smallest reduction in modulus, while PP5 exhibited the lowest decline in yield strength. This behavior can be attributed to the closed-cell structure formed during processing, which results in a characteristic cellular morphology. Moreover, the relatively smaller cell sizes are believed to have mitigated the extent of mechanical degradation. Zeng et al. similarly reported that tensile strength tends to increase with decreasing cell size, highlighting the strong dependence of mechanical performance on cellular microstructure.^91^ A noticeable decrease in impact strength was also observed after the foaming process. This phenomenon can be attributed to the presence of micro voids and cellular interfaces within the polymer matrix, which act as localized stress concentrators. The reduction in impact strength was particularly pronounced in PP5 and PP7, indicating that cellular morphology parameters such as cell size distribution and wall thickness adversely affect the material’s energy absorption capability. Although the incorporation of LDPE and reactive components such as TMPTA and DHBP promotes chain branching and enhances ductility, an inhomogeneous cellular structure may counteract these benefits by facilitating crack propagation and reducing energy dissipation efficiency. Therefore, the pronounced loss of toughness observed in PP5 and PP7 highlights the complex interplay between cell morphology and chain branching density. These findings emphasize that optimizing foaming parameters and reactive additive concentrations is essential to achieving an optimal balance between lightweight design and mechanical integrity in polypropylene-based foam systems.

**Figure 21:**
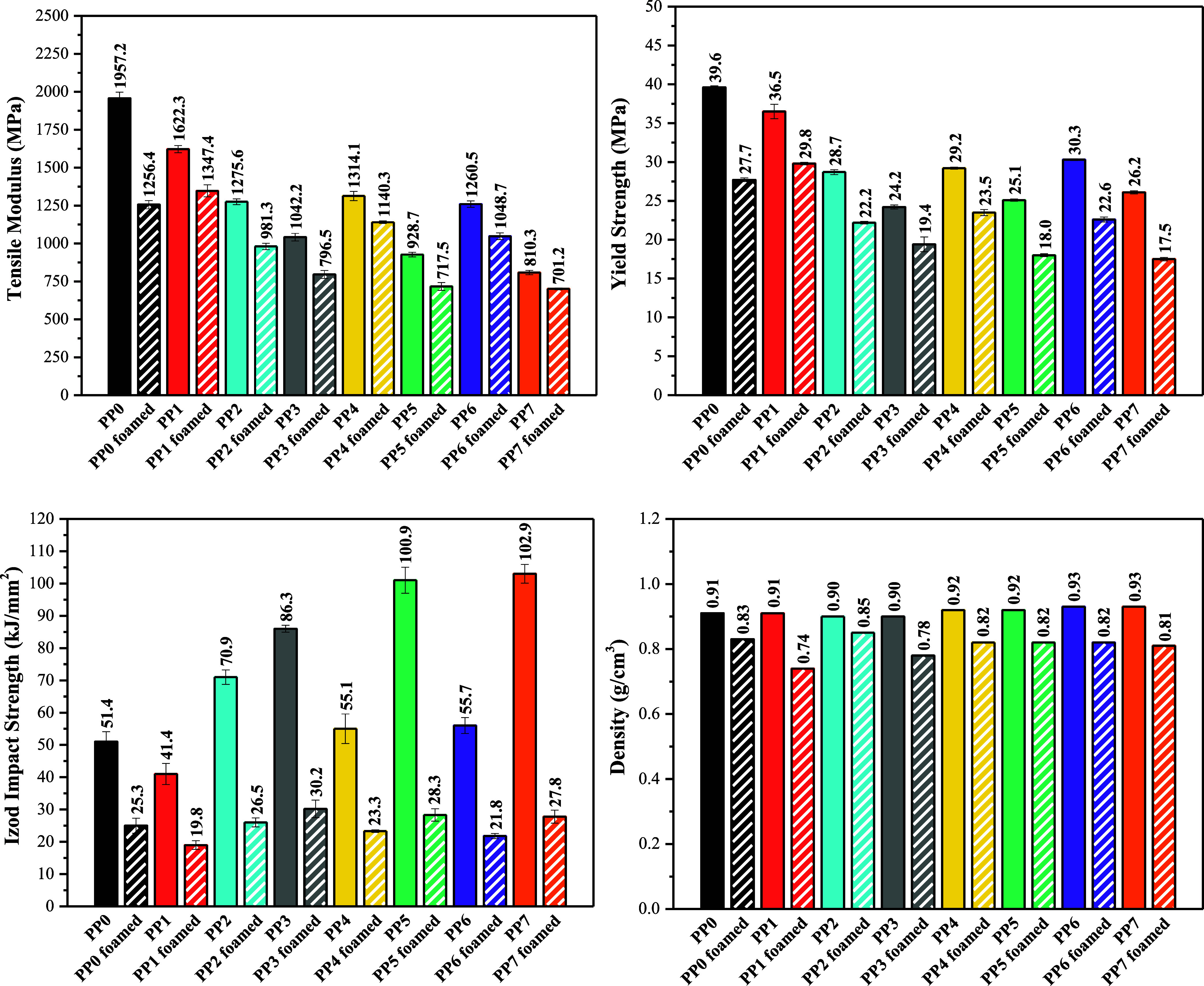
Mechanical properties of PP, PP/LDPE blends, and their foamed counterparts.

Foaming resulted in a general drop in the mechanical properties for all the recycled polypropylene (rPP) systems, as would be predicted by the introduction of voids and lower effective load-bearing cross-sectional area.^89^ rPP0 had the best retention of stiffness among the recycled systems, and the tensile modulus reduced from only 1457 to 1348 MPa upon foaming (Figure 22). Yet, its yield strength reduced by 32.2 to 27.6 MPa and the Izod impact strength reduced by half 43.7 to 24.7 kJ/m^2^. This shows that whereas rPP0 maintains rigidity, the extensive and relatively sparse cells reduce toughness.^92,93^ rPP5 had the most balanced mechanical behavior. The modulus reduced less than rPP0, and the highest impact strength for the recycled systems, which is before foaming 100.4 to 33.5 kJ/m^2^ after foaming were recorded by it. The finer cellular structure seen in the rPP5 foams improves energy absorption and allows it to retain more toughness than rPP0. rPP7 exhibited intermediate behavior. The modulus reduced by 796 to 615 MPa upon foaming and the impact toughness reduced from 94.3 to 28.4 kJ/m^2^. Its behavior parallels the intermediate cell density and relatively larger cell size than rPP5 and shows excessive branching/cross-linking at the expense of property retention even though excellent melt strength is achieved by them. These findings verify that foaming inevitably lowers the stiffness and strength in recycled PP; however, the degree thereof heavily depends on the foam morphology. rPP0, while stiff, goes brittle because the cell densities are low. rPP5 optimizes the best compromise and retains higher toughness after foaming, whereas rPP7 shows lower property retention than rPP5. The findings reveal that a balanced branching degree of rPP5 is best for attaining a balance between toughness and stiffness in recycled foams of polypropylene.^88^

**Figure 22:**
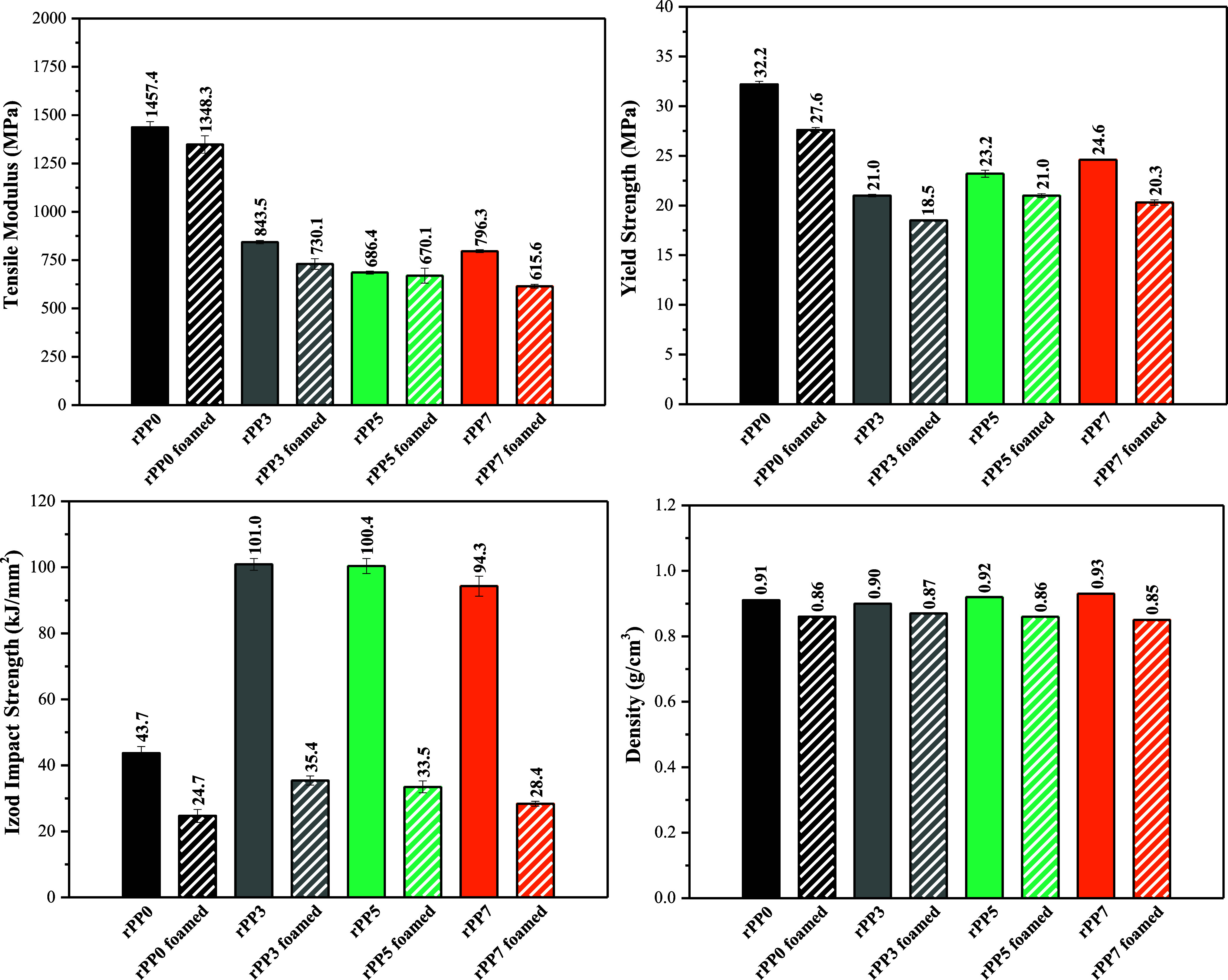
Mechanical properties of rPP, rPP/LDPE blends, and their foamed counterparts.

## Conclusions

This work demonstrated a commercially viable reactive extrusion method to produce high melt strength polypropylene from virgin and recycled polypropylene and LDPE. Concomitant application of peroxide (DHBP) and polyfunctional coagent (TMPTA) played an important role in the structure modification and end-use properties of the blends. TMPTA addition within the concentration range from 2 to 5 wt % helped mitigate chain scission commonly triggered by peroxide and caused an observable melt strength and elasticity gain. Such an improvement was manifest by increased complex viscosity, enhanced storage modulus (*G*′), and an observable shear-thinning nature, all common signs of long-chain branched architectures. Besides that, the Cole–Cole plots also provided further evidence consistent with the presence of long relaxation modes corresponding to the formation of branching. FTIR spectroscopy indicated the absence of chemical interactions among the components under the investigated conditions. SEM analysis revealed that reactive modification refined the LDPE domain size and improved interfacial adhesion between PP and LDPE phases, leading to a more compatibilized morphology. Better melt elasticity led to higher foamability. The blend including 40 wt % LDPE and 2 wt % TMPTA, PP5, provided a similar microcellular structure with the maximum cell density (1.1 × 10^8^ cells/cm^3^) and minimum mean cell size (7.48 μm). This highlights the significance of obtaining the optimum value of the degree of branching, not the maximum value, to obtain the most favorable foam morphology within the investigated formulation range. Another important finding is the successful upcycling of the reactive modification process for recycled PP. rPP5 blend exhibited comparable foamability and mechanical properties to virgin material and provided an economical method to convert industrial waste to high-performance foamed materials. Overall, the research provides a comprehensive strategy for producing polypropylene foam with high melt strength in an environmentally friendly manner. By defining correlations observed in this study between reactive modification, rheological properties, and foaming characteristics, the research suggests a scalable approach to expand the potential of virgin and recycled polyolefins to more complex engineering end-use applications, namely lightweight automotive components, energy-efficient insulating materials, and eco-friendly packaging materials.
